# Duplex Labeling and Manipulation of Neuronal Proteins Using Sequential CRISPR/Cas9 Gene Editing

**DOI:** 10.1523/ENEURO.0056-22.2022

**Published:** 2022-07-26

**Authors:** Wouter J. Droogers, Jelmer Willems, Harold D. MacGillavry, Arthur P. H. de Jong

**Affiliations:** Cell Biology, Neurobiology and Biophysics, Department of Biology, Faculty of Science, Utrecht University, 3584 CH, Utrecht, The Netherlands

**Keywords:** CRISPR/Cas9, fluorescence microscopy, knock-in, multiplex genome editing, neurons

## Abstract

CRISPR/Cas9-mediated knock-in methods enable the labeling of individual endogenous proteins to faithfully determine their spatiotemporal distribution in cells. However, reliable multiplexing of knock-in events in neurons remains challenging because of cross talk between editing events. To overcome this, we developed conditional activation of knock-in expression (CAKE), allowing efficient, flexible, and accurate multiplex genome editing. To diminish cross talk, CAKE is based on sequential, recombinase-driven guide RNA (gRNA) expression to control the timing of genomic integration of each donor sequence. We show that CAKE is broadly applicable in rat neurons to co-label various endogenous proteins, including cytoskeletal proteins, synaptic scaffolds, ion channels and neurotransmitter receptor subunits. To take full advantage of CAKE, we resolved the nanoscale co-distribution of endogenous synaptic proteins using super-resolution microscopy, demonstrating that their co-organization correlates with synapse size. Finally, we introduced inducible dimerization modules, providing acute control over synaptic receptor dynamics in living neurons. These experiments highlight the potential of CAKE to reveal new biological insight. Altogether, CAKE is a versatile method for multiplex protein labeling that enables the detection, localization, and manipulation of endogenous proteins in neurons.

## Significance Statement

Accurate localization and manipulation of endogenous proteins is essential to unravel neuronal function. While labeling of individual proteins is achievable with existing gene editing techniques, methods to label multiple proteins in neurons are limiting. We introduce a new CRISPR/Cas9 strategy, CAKE, achieving faithful duplex protein labeling using sequential editing of genes. We use CAKE to visualize the co-localization of essential neuronal proteins, including cytoskeleton components, ion channels and synaptic scaffolds. Using super-resolution microscopy, we demonstrate that the co-organization of synaptic scaffolds and neurotransmitter receptors scales with synapse size. Finally, we acutely modulate the dynamics of synaptic receptors using labeling with inducible dimerization domains. Thus, CAKE mediates accurate duplex endogenous protein labeling and manipulation to address biological questions in neurons.

## Introduction

The spatiotemporal distribution of proteins dictates virtually all functions of cells, and the accurate detection of endogenous proteins is an essential strategy in cell biological research (Choquet et al., 2021). Because protein overexpression and antibody labeling have significant limitations in accuracy and specificity, there is a pressing need to develop novel techniques that detect endogenous proteins in biological preparations. Recent CRISPR/Cas9-based genome editing methods have addressed this need by inserting (fluorescent) tags in specific genes, creating knock-ins, and now make it possible to reliably detect endogenous protein distribution with fluorescence microscopy in a wide variety of biological preparations (Auer et al., 2014; Nakade et al., 2014; Mikuni et al., 2016; Schmid-Burgk et al., 2016; Suzuki et al., 2016; Artegiani et al., 2020). However, simultaneous labeling of multiple protein species in individual cells remains challenging with commonly used genome editing methods, particularly in postmitotic cells such as neurons. We reasoned that such genetic tools are mandatory to study the co-distribution of proteins, and would present an elegant approach to manipulate the distribution and dynamics of endogenous proteins.

The fact that neurons are postmitotic cells severely complicates both simplex and multiplex genome editing strategies: it prevents the isolation and expansion of desired clones to create isogenic cell lines and precludes multiple independent rounds of gene modification. Furthermore, insertion of the donor DNA using the highly accurate homology-directed repair (HDR) pathway predominantly occurs in the S/G_2_ phases of mitosis (Orthwein et al., 2015), and is strongly disfavored in nondividing cells. While successful genomic insertion of epitope tags using HDR in neurons has been reported (Nishiyama et al., 2017; Matsuda and Oinuma, 2019), most neuronal knock-in methods instead use the more efficient, but error-prone nonhomologous end joining (NHEJ) mechanism which remains active in postmitotic cells. Most NHEJ-based methods target the coding sequence of genes (Schmid-Burgk et al., 2016; Suzuki et al., 2016; Gao et al., 2019; Willems et al., 2020), but more recent strategies replace endogenous exons (Danner et al., 2021; Fang et al., 2021) or introduce novel exons in intronic sequences (Zhong et al., 2021) to mitigate the effects of indel mutations. Indels can also be reduced in neurons using microhomology-mediated end joining (MMEJ; Yao et al., 2017).

**Figure 1.**
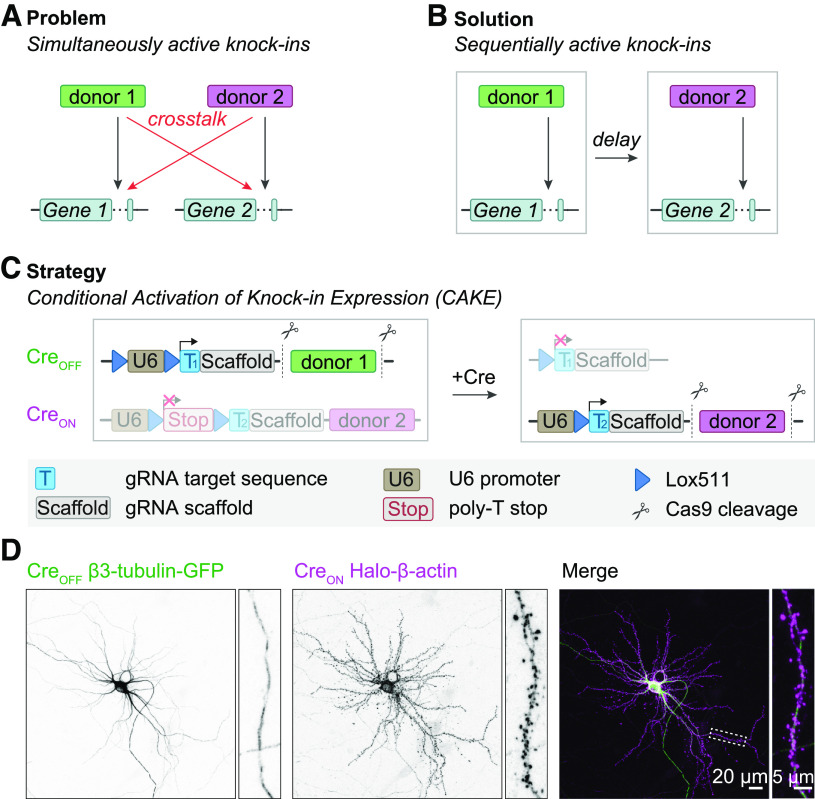
Multiplex labeling of endogenous proteins using CAKE. ***A***, Illustration of the problem of multiplex knock-in strategies based on NHEJ. With simultaneous editing of multiple genes, donor DNAs can be integrated in either allele, leading to cross talk. See Extended Data Figure 1-1*F* for an example of an incorrect knock-in. ***B***, Proposed solution for multiplex knock-ins. By introducing a delay between two genome editing events, cross talk can be avoided. CAKE is designed to control the delay between the two events, using Cre-recombinase or Flp-recombinase. ***C***, CAKE strategy. The Cre_OFF_ vector is active in the absence of Cre, leading to editing of the first target gene, and removal of the Cre_OFF_ donor for subsequent genomic integration. Upon addition of Cre, gRNA expression from Cre_OFF_ is deactivated, and gRNA expression from Cre_ON_ vector is enabled for editing the second target gene. See Figure 4 for example methods to deliver active Cre. All donors contain a fluorophore or epitope tag flanked by a protospacer adjacent motif (PAM) and target sequence (data not shown; see Extended Data Fig. 1-3*B* and Willems et al., 2020). See Extended Data Figure 1-4 for step-by-step guidelines for making knock-ins. ***D***, Example confocal image of a Cre_OFF_ β3-tubulin-GFP and Cre_ON_ Halo-β-actin knock-in; 20-μl lenti-Cre was added at DIV 7, and cells were fixed at DIV 14. See Extended Data Figure 1-2 for low-magnification examples of single and double knock-in cells.

Although these methods faithfully label individual proteins in neurons, multiplex epitope tagging using CRISPR/Cas9 has remained challenging. While achievable with HDR, its efficacy is generally too low for routine use (Mikuni et al., 2016; Matsuda and Oinuma, 2019). NHEJ in turn, operates without homology between donor DNA and target locus, and therefore the donor DNA can indiscriminately integrate in any double-stranded break (DSB), leading to a high degree of donor integration in the incorrect locus (i.e., cross talk; Fig. 1*A*). Gao et al. (2019) circumvented this problem by creating donor DNAs that prevent protein labeling when inserted in the incorrect gene. This strategy successfully generated double knock-ins, but has restrictions on the location of the protein tag, and indels or integration of the incorrect donor may generate null mutations (Gao et al., 2019).

We reasoned that cross talk between donor DNAs could be diminished by separating genome editing events in time (Fig. 1*B*; also see Chylinski et al., 2019). Using our NHEJ-based Open Resource for the Application of Neuronal Genome Editing (ORANGE) toolbox we previously achieved this for a small number of genes with a mechanism we dubbed conditional activation of knock-in expression (CAKE; Willems et al., 2020). In this approach, a GFP-2A-Cre donor sequence was fused to the first gene, which after successful knock-in switched on the expression of the second knock-in vector (Extended Data Fig. 1-1*A*). However, we did observe cross talk between the loci for some knock-in combinations, suggesting insufficient control over the delay between the two genome editing events (Willems et al., 2020).

To overcome this, we implemented major improvements of our CAKE strategy that result in reproducible multiplex genome editing in neuronal preparations. We demonstrate that, with Cre-dependent knock-in vectors and precise timing of Cre-recombinase activation, a high rate of correct double knock-in cells can be attained, while strongly diminishing cross talk. Furthermore, we applied CAKE to study and manipulate the positioning of multiple endogenous proteins simultaneously in individual cells, illustrating that CAKE is a versatile method to resolve complex biological questions.

## Materials and Methods

### Ethics statement

All experiments were approved by the Dutch Animal Experiments Committee (Dier Experimenten Commissie; DEC; AVD1080020173404), performed in line with institutional guidelines of Utrecht University, and conducted in agreement with Dutch law (Wet op de Dierproeven, 1996) and European regulations (Directive 2010/63/EU). Timed pregnant Wistar rats were obtained from Janvier Labs.

### Molecular cloning

Constructs were made using standard laboratory techniques. All CAKE knock-in backbones are numbered as pORANGE CAKE (pOCx; see Extended Data Fig. 1-3*A* and Table 1 for a complete overview). For most pOCx vectors, variants with and without CAG SpCas9 were created. All knock-ins used in this study are listed in Table 2. Design rationales of all new knock-ins are found in Table 3. All primer sequences are found in Table 4.

**Table 1:** Vectors

Vector	Purpose	Source	Addgene ID
pOC1 cloning template vector	Cre_ON_ knock-in vector	This study	183420
pOC2 cloning template vector	Cre_OFF_ knock-in vector	This study	183421
pOC3 cloning template vector	Cre_ON_ Flp_OFF_ knock-in vector	This study	183422
pOC4 cloning template vector	Flp_ON_ knock-in vector with FLAG-SpCas9 expression	This study	183423
pOC5 cloning template vector	Flp_OFF_ knock-in vector	This study	183424
pFUGW NLS-Cre	Lentiviral Cre vector	This study	183425
pFUGW GFP-NLS-Cre	Lentiviral GFP-Cre vector	Kaeser et al. (2011)	n/a
pFUGW NLS-FlpO	Lentiviral FlpO vector	This study	183448
pCAG ERT2 Cre ERT2	Tamoxifen-inducible Cre	Matsuda and Cepko (2007)	13777
pCAG ERT2 Cre ERT2 lox	Cre_OFF_ tamoxifen-inducible Cre	This study	183447
pOC1 Halo-*Actb*	Cre_ON_ knock-in for Halo-β-actin	This study	183426
pOC1 *Gria1-*HA	Cre_ON_ knock-in for GluA1-HA	This study	183427
pOC1 *Gria1-*GFP-FRB	Cre_OFF_ knock-in for GluA1-GFP-FRB	This study	183428
pOC2 GFP-*Actb*	Cre_OFF_ knock-in for GFP-β-actin	This study	183429
pOC2 *Gria1-*GFP	Cre_OFF_ knock-in for GluA1-GFP	This study	183430
pOC2 *Tubb3* no donor	Cre_OFF_ gRNA expression for β3-tubulin	This study	183431
pOC2 *Tubb3-*GFP	Cre_OFF_ knock-in for β3-tubulin-GFP	This study	183432
pOC2 *Gria1-*Halo	Cre_OFF_ knock-in for GluA1-Halo	This study	183433
pOC2 GFP-*Mpp2*	Cre_OFF_ knock-in for GFP-MPP2	This study	183434
pOC2 *Dlg4-*Halo-FKBP	Cre_OFF_ knock-in for PSD95-Halo-FKBP	This study	183435
pOC3 smFP-HA-*Cacna1e*	Cre_ON_ Flp_OFF_ knock-in for smFP-HA-CaV2.3	This study	183436
pOC4 mRuby3-*Actb*	Flp_ON_ knock-in for mRuby3-β-actin	This study	183437
pOC4 smFP-myc-*Kcnn2*	Flp_ON_ knock-in for smFP-myc-SK2	This study	183438
pOC4 *Grin1-*smFP-V5	Flp_ON_ knock-in for GluN1-smFP-V5	This study	183439
pOC5 *Tubb3-*GFP	Flp_OFF_ knock-in for β3-tubulin-GFP	This study	183440
pOC5 smFP-HA-*Cacna1e*	Flp_OFF_ knock-in for smFP-HA-Ca_V_2.3	This study	183441
pOC5 *Kcnma1-*smFP-HA	Flp_OFF_ knock-in for BK-smFP-HA	This study	183442
pORANGE *Tubb3-*GFP	GFP knock-in for β3-tubulin-GFP (1× donor)	Willems et al. (2020)	131497
pORANGE *Tubb3-2*×GFP	GFP knock-in for β3-tubulin-GFP (2× donor)	This study	183443
pORANGE *Tubb3-3*×GFP	GFP knock-in for β3-tubulin-GFP (3× donor)	This study	183444
pORANGE *Tubb3-4*×GFP	GFP knock-in for β3-tubulin-GFP (4× donor)	This study	183445
pORANGE *Tubb3-*GFP-2A-Cre	GFP-2A-Cre knock-in for β3-tubulin-GFP	Willems et al. (2020)	n/a
pTubb3 MC	Minicircle GFP donor for β3-tubulin	Suzuki et al. (2016)	87112
pOC2 CRISPIE-Halo-*Homer1*	Cre_ON_ knock-in for CRISPIE-Halo-Homer1	This study	183446
pOC2 *Dlg4*-Halo	Cre_OFF_ knock-in for PSD95-Halo	This study	183449

All vectors that were used in this study are listed with their name, purpose, source and Addgene ID

**Table 2:** CRISPR knock-ins

Gene	Protein	Target sequence (PAM is underlined)	Site of integration (in or before amino acid)	MIT score (in rat)	Conservation of target sequence in mouse	Source
*Actb*	β-actin	TGTGCCTTGATAGTTCGCCA TGG	1 bp before ATG	84	1 mismatch	Willems et al. (2020)
*Cacna1e*	Ca_V_2.3 (R-type)	CAGGATGGCTCGCTTCGGGGAGG	G5	88	Yes	Willems et al. (2020)
*Dlg4*	PSD95	AATCAGAGTCTCTCTCGGGCTGG	R721	80	Yes	Willems et al. (2020)
*Gria1*	GluA1	GGGAGCCACAGGATTGTAAC TGG	STOP codon	72	Yes	Willems et al. (2020)
*Homer1*	Homer1	TATATGCCTCCGGTGCCCAG AGG	G2	49	Yes	This study
*Kcnma1*	BK	GAACGTACTTCTGTTTGTCGGGC	D1201	86	Yes	This study
*Kcnn2*	SK2	TAGCTACTCTCAGATGAAGT TGG	T571	68	1 mismatch	This study
*Mpp2*	MPP2	TCAGAGTTCGTGGCAGCAAC CGG	A4	74	1 mismatch	This study
*Tubb3*	β3-tubulin	GCTGCGAGCAACTTCACTTGGG	STOP codon	59	Yes	Willems et al. (2020)
*Grin1*	GluN1	CTTGGGGTCGCAGGCGGCGC TGG	A20	85	Yes	Willems et al. (2020)

All knock-ins that were used in this study are listed with their gene and protein name, target sequence and target properties.

**Table 3:** Design rationale for new knock-ins

Gene	Protein	Endogenous location	Tag location	Design rationale
*Homer1*	Homer1	Enriched in dendritic spines and PSD (Sala et al., 2001)	N terminus	The tag is inserted at the second amino acid of Homer1, thereby minimizing effects on functional domains.
*Kcnma1*	BK	Punctate distribution in axons, dendrite and soma (Sailer et al., 2006)	C terminus	C-terminal addition of fluorophore does not affect localization or function of BK (Giraldez et al., 2005)
*Kcnn2*	SK2	Punctate distribution in axons and dendrites (Ballesteros-Merino et al., 2012; Sun et al., 2015)	C terminus	C-terminal tagging does not appear to affect SK-channel function (Yang et al., 2017)
*Mpp2*	MPP2	Punctate signal in dendrites; enriched in dendritic spines but absent from PSD (Kim et al., 2016; Rademacher et al., 2016)	N terminus	N-terminal, but not C-terminal, sequences are present in all splice variants; N-terminal tagging does not disrupt protein expression (Baumgartner et al., 2009)

For each knock-in made in this study, the gene and protein name, endogenous location, tag location and design rationale are listed.

**Table 4:** Primers

Name	Sequence	Purpose
AJ19164	CTAGGACGCGTTAACTTACGGTAAATGGCCCGGC	Removal of Cas9 from pORANGE LOX
AJ19165	GGCCGCCGGGCCATTTACCGTAAGTTAACGCGTC	Removal of Cas9 from pORANGE LOX
AJ19069	TTGCTCACATGTATAACTTCGTATAGTATAAATTATACGAAGTTATGATCCGACGCCGCC	Clone pOC2 Cre_OFF_
AJ20047	TATCTTCTTGTACACGAAGACAAACAAGGCTTATAACTTCGTATAATTTATACTATACGAAGTTATACTAACTTTACAGT	Clone pOC2 Cre_OFF_
AJ20116	GGCGGCCGCAAAAAAAAAAAGAAGTTCCTATACTTTCTAGAGAATAGGAACTTCCAGCATAGCTCTTAAACAGGTCTTCTCG	Clone pOC4 Flp_ON_
AJ20117	CAGAATTCTGGCCTGCAGGGAAGTTCCTATTCTCTAGAAAGTATAGGAACTTCCAGCATAGCAAGTTTAAATAAGGCTAGTCCGTTATC	Clone pOC4 Flp_ON_
AJ20120	AAAGTATAGGAACTTCCAGCATAGCAAGTTAAAATAAGGCTAGTCCGTTATC	Clone pOC3 Cre_ON_ Flp_OFF_; pOC4 Flp_ON_
AJ20121	CCTCGAGTCGACAATTGCTAGCAAGC	Clone pOC3 Cre_ON_ Flp_OFF_; pOC4 Flp_ON_
AJ20122	CGGCCTTTTTACGGTTCCTGG	Clone pOC4 Flp_ON_
AJ20124	TGCTGGCCTTTTGCTCACATGTGAAGTTCCTATTCTCTAGAAAGTATAGGAACTTCGATCCGACGCCGCCATC	Clone pOC3 Cre_ON_ Flp_OFF_
AJ20125	GCTGGAAGTTCCTATACTTTCTAGAGAATAGGAACTTCCAGCATAGCTCTAAAACAGGTCTTCTTG	Clone pOC3 Cre_ON_ Flp_OFF_
AJ20126	GGTGCTTGTCAGTTCCTCTGAGG	Clone pOC3 Cre_ON_ Flp_OFF_
AJ20127	CGATTTTTGAATTCGTTCCTCAGAGGAAC	Clone pOC3 Cre_ON_ Flp_OFF_
AJ20128	ATACTATCTAGAACAACCATGGGCAAGAAGAAGAGGAAGGTGTCCAATTTAC	Clone pFUGW-NLS-Cre
AJ20129	ATTGTTAACGGATCCGCTAGCC	Clone pFUGW-NLS-Cre
AJ20130	ATACTTTCTAGAGCCGCCACCATGGCTCC	Clone pFUGW-NLS-Flp^O^
AJ20131	ATACAAGCTAGCTCAGATCCGCCTGTTGATG	Clone pFUGW-NLS-Flp^O^
AJ21030	TGGAAAGGACGAAACACCGGGTCTTCGAGAAGACCTGTTTTAGAGCTATGCTGG	Clone pOC5 Flp_OFF_
AJ21031	TTGCTAGCAAGCTTCTAGAAAAAAAAAAAAGAAGTTCCTATACTTTCTAGAGAATAGGAACTTCGAATTGGCGCACGCG	Clone pOC5 Flp_OFF_
AJ21032	CGAAGACCCGGTGTTTCGTCC	Clone pOC5 Flp_OFF_
AJ21053	GTCTCATCATTTTGGCAAAGGCTAGCATAACTTCGTATAGCATACATTATACGAAGTTATGAATTCCCGGGTGAGCCGCCA	Clone pCAG ER^T2^-Cre-ER^T2^ lox
AJ21054	TTTCCCTGCCACAGCTTGATAGCGGCCGCATAACTTCGTATAGCATACATTATACGAAGTTATGGCCGCACTCCTCAGGTGCAGGC	Clone pCAG ER^T2^-Cre-ER^T2^ lox
WD0116	ATAAAGCTTTATATGCCTCCGGTGCCCAGGGGGAGCCAGGGAATAAGACG	Clone pOC2 CRISPIE-Halo-Homer1
WD0117	ATAACGCGTCCCCTGGGCACCGGAGGCATATAGAATAATATCATAGCATCC	Clone pOC2 CRISPIE-Halo-Homer1
JW381	CACGCTCGAGATTTCCGGCAGCGGAGGCAGCGCTGGAGTGCAGGTGGAAACCAT	Clone pOC2 PSD95-Halo-FKBP
JW382	GAGACTCTGATTTAGCGTCGACTCCTTCCAGTTTTAGAAGCTCCACATCGAA	Clone pOC2 PSD95-Halo-FKBP
JW375	GAGCTGTACAGCGCTTCTGGTGGTGGTAGCTACGT	Clone pOC1 GluA1-GFP-FRB
JW376	CAGGATTGTAACGGGAGCAGTCGACTCACTTTGAGATTCGTCGGAACACAT	Clone pOC1 GluA1-GFP-FRB

All primers used to make the vectors that were constructed in this study.

The Cre_ON_ knock-in vector (pOC1, Addgene #183420) is based on pORANGE LOX from (Willems et al., 2020, Addgene #139651), where CAG HA-SpCas9 was removed with XmaJI and NotI and replaced with primers AJ19164 and AJ19165 using primer ligation.

To clone the Cre_OFF_ knock-in vector (pOC2, Addgene #183421), lox-U6-lox was created by PCR from pOC1 with primers AJ19169 and AJ20047, digested with PscI and Bsp1407I, and ligated into pOC1 that was digested with PscI and Bsp1407I as well.

The Cre_ON_ Flp_OFF_ knock-in vector (pOC3, Addgene #183422) was based on pOC1, where one Frt site was cloned into the guide RNA (gRNA) scaffold (Chylinski et al., 2019) and one before the U6 promoter. PCR fragments were obtained with primers AJ20124–AJ20126, AJ20127–AJ20125, and AJ20120–AJ20121 using pOC1 as template. pOC1 was digested with NheI and PscI, and all fragments were ligated using HiFi assembly (New England Biolabs).

The Flp_ON_ knock-in vector (pOC4, Addgene #183423) was designed analogous to Chylinski et al., 2019. PCR fragments were created using primers AJ20120–AJ20121 with pOC1 as template, and primers AJ20122–AJ20116 and AJ20117–AJ20121 with pORANGE empty (Addgene #131471; Willems et al., 2020) as template. pORANGE was digested with PscI and NheI, and all fragments were ligated with HiFi assembly.

The Flp_OFF_ knock-in vector (pOC5, Addgene #183424) was designed similar as in Chylinski et al. (2019). PCR fragments were obtained with primers AJ21030–AJ21031 with pOC3 as template, and AJ20122 and AJ21032 with pOC4 as template. pOC3 was digested with PscI and HindIII and fragments were ligated with HiFi assembly.

To obtain pORANGE β3-tubulin-2xGFP (Addgene #183443), the donor DNA from pORANGE β3-tubulin-GFP (Willems et al., 2020) was isolated using XmaJI and XbaI, and ligated in the XbaI site of pORANGE β3-tubulin-GFP. Subsequently, to create pORANGE β3-tubulin with 3xGFP (Addgene #183444) or 4×GFP (Addgene #183445), the pORANGE β3-tubulin-2xGFP double donor DNA was isolated with XmaJI and XbaI, and ligated in the XbaI site of pORANGE β3-tubulin-GFP or pORANGE β3-tubulin-2xGFP, respectively. Because of the repeated sequences, the orientation of the inserts could not be confirmed, but this should not affect performance of the knock-in. pFUGW-NLS-Cre (Addgene #183425) was created using a PCR reaction for NLS-Cre with primers AJ20128–AJ20129 using pFUGW-GFP-NLS-Cre (a gift from Pascal Kaeser, Harvard Medical School, Boston MA; Kaeser et al., 2011) as template. The PCR product was digested with NheI and XbaI, and ligated in NheI and XbaI sites of pFUGW. pFUGW-NLS-Flp^O^ (Addgene #183448) was created using a PCR reaction for NLS-Flp^O^ with primers AJ20130–AJ20131 using pAAV hSynapsin Flp^O^ (Addgene #60663), a gift from Massimo Scanziani (Xue et al., 2014), as template. The PCR product was digested with NheI and XbaI, and ligated in NheI and XbaI sites of pFUGW. pCAG ER^T2^-Cre-ER^T2^ lox (Addgene #183447) was created by digesting pCAG ER^T2^-Cre-ER^T2^ (Matsuda and Cepko, 2007) with EcoRI and NotI. Both resulting DNA fragments were mixed with primers AJ21053 and AJ21054, and ligated with HiFi assembly.

All knock-ins were based on ORANGE (Suzuki et al., 2016; Willems et al., 2020) and cloned in pOCx backbones as described in Extended Data Figure 1-3*B*. For a complete description of knock-in design and cloning, see Extended Data Figure 1-4. gRNA target sequences can be found in Table 2. Fluorophores and epitope tags in the donor DNAs were exchanged using universal BmtI and AfeI restriction sites that are present in linker sequences surrounding the fluorophore. Spaghetti monster fluorescent proteins (smFP) were obtained from Addgene #59 759 (HA) and #59 758 (V5), a gift from Loren Looger (Viswanathan et al., 2015).

For Rapalog-inducible assays, FKBP (primer JW381 ad JW382) and FRB (primer JW375 and JW376) were amplified using PCR (both a gift from Lukas Kapitein; Kapitein et al., 2010a). These fragments were cloned into the AfeI site of pOC2 PSD95-Halo (Addgene #183449) and pOC1 GluA1-GFP (Addgene #183430), respectively, using HiFi assembly.

The design of pOC2 CRISPIE Halo-Homer1 (Addgene #183446) was adapted from the strategy of Zhong et al. (2021) and described in Extended Data Figure 2-1. The donor DNA was ordered as a gBlock from IDT and PCR amplified using WD0116 and WD0117. Donor consists of Halo flanked by the splicing acceptor and donor of exon 8 of *Septin 3* (Extended Data Fig. 2-1). Splicing acceptor and donor include 100-bp intronic and 10-bp exonic sequences. Target sequences were selected using CRISPOR and the BROAD institute sgRNA designer (Doench et al., 2016; Concordet and Haeussler, 2018).

### Antibodies and reagents

The following primary antibodies were used in this study: mouse anti-GFP 1:2000 dilution (Thermo Fisher Scientific, RRID: AB_221568), rabbit anti-GFP 1:2000 (MBL, RRID: AB_591819), rabbit anti-Halo 1:1000 (Promega, RRID: AB_713650), rat anti-HA 1:500 (Sigma-Aldrich, RRID: AB_390919), and mouse anti V5 1:1000 (Invitrogen, RRID: AB_2556564). Alexa488-, Alexa568-, and Alexa647-conjugated secondary antibodies were used at 1:400 dilution, obtained from Life Technologies. CF568-conjugated secondary antibodies were used at 1:600 dilution, obtained from Sigma-Aldrich. Molecular biology reagents were obtained from Thermo Fisher Scientific. GFP minicircle for β3-tubulin were custom made at System Biosciences, produced from pTubb3 MC (Addgene #87112, Suzuki et al., 2016). 4OH-tamoxifen was acquired from Sigma-Aldrich (catalog #H7904), and kept at 20 mm in ethanol in single use aliquots at −20°C. For Halo labeling, Halo Ligands JF549 (Promega, GA1110) and JF646 (Promega, GA1121) were used (Grimm et al., 2015). Both dyes were dissolved using DMSO to 0.2 mm on arrival. Working concentration was 0.2 μm (1:1000). For FKBP-FRB dimerization, we used Rapalog (TaKaRa, #635057).

### Dissociated neuronal cultures

Dissociated hippocampal cultures were prepared from embryonic day (E)18 rat brains of both genders (Kapitein et al., 2010b). Dissociated neurons were plated on Ø18-mm coverslips coated with poly-L-lysine (37.5 μg/ml, Sigma-Aldrich) and laminin (1.25 μg/ml, Roche Diagnostics) at a density of 100,000 neurons per well, in Neurobasal medium (NB), supplemented with 1% penicillin and streptomycin (pen/strep), 2% B27, and 0.5 mm L-glutamine (all from Invitrogen; NB-complete medium) at 37°C in 5% CO_2._ From day *in vitro* (DIV) 1 onwards, medium was refreshed every 7 d by replacing half of the medium with Brainphys neuronal medium supplemented with 2% NeuroCult SM1 neuronal supplement (Stem Cell Technologies) and 1% pen/strep (BP-complete medium).

### Transfection of dissociated hippocampal neurons

Neurons were transfected at DIV 1–3 using Lipofectamine 2000 (Invitrogen). For one Ø18-mm coverslip seeded with 100,000 neurons, up to 2-μg DNA was used, which typically results in a few hundred to one thousand transfected cells per coverslip. DNA concentrations were determined using Nanodrop. For CAKE double knock-ins, the mixture contained 3.9–197 fmol (10–500 ng) Cre_OFF_ knock-in, 3.6–178 fmol (10–500 ng) Cre_ON_ knock-in, and 171 (500 ng) pCAG FLAG-SpCas9 expression. Experiments with inducible Cre used 2–20 fmol (10–100 ng) pCAG ERT2-Cre-ERT2 (Addgene #3777, Matsuda and Cepko, 2007) per coverslip. DNA was mixed with 3.3-μl Lipofectamine in 200-μl NB medium and incubated for 30 min at room temperature (RT). Next, 500-μl conditioned medium was transferred to a new culture plate and replaced by 300-μl NB supplemented with 0.5 mm L-glutamine. The DNA/Lipofectamine mix was added to the culture and incubated at 37°C and 5% CO_2_. After 90–120 min, coverslips were transferred to the new culture plate with conditioned medium and 500 μl new BP-complete and kept at 37°C and 5% CO_2_ for between 1 and 20 d, depending on the experiment.

### Immunocytochemistry

Hippocampal neurons were fixed at DIV 14–23 in 80 mm PIPES, 1 mm EGTA, 2 mm MgCl_2_ pH 6.8, and 4% paraformaldehyde (Electron Microscopy Sciences), for 5–10 min at 37°C and washed three times in PBS containing 0.1 m glycine (PBS/Gly). Neurons were blocked and permeabilized in blocking buffer [10% (v/v) normal goat serum (NGS; Abcam) in PBS/Gly with 0.1% (v/v) Triton X-100] for 1 h at 37°C. Next, coverslips were incubated with primary antibodies diluted in incubation buffer [5% (v/v) NGS in PBS/Gly with 0.1% (v/v) Triton X-100] for 2 h at RT or overnight at 4°C, depending on the antibodies used. Coverslips were washed three times with PBS/Gly and incubated with secondary antibodies diluted 1:400 in incubation buffer for 1 h at RT. Coverslips were washed three times in PBS/Gly, dipped in milliQ water (MQ), and mounted in Mowiol mounting medium (Sigma-Aldrich).

### Lentivirus production

For lentivirus production, HEK293T cells were maintained at a high growth rate in DMEM (Lonza) supplemented with 10% fetal calf serum (Corning) and 1% pen/strep. One day after plating on 10-cm dishes, cells at ∼70% confluency were transfected using polyethylenimine (PEI; Polysciences) with second-generation lentiviral packaging vectors (psPAX2 and 2MD2.G) and pFUGW-NLS-Flp^O^, pFUGW-NLS-Cre or pFUGW-GFP-NLS-Cre at a 1:1:1 molar ratio. At 6 h after transfection, cells were washed once with PBS, and medium was replaced with 10 ml DMEM containing 1% pen/strep. At 48 h after transfection, the supernatant was harvested, centrifuged 5 min at 700 × *g* to remove cell debris, and stored in aliquots at −80°C until use. Cultured hippocampal neurons were infected at DIV 3–9 with 20-μl virus added per well, unless indicated otherwise.

### Quantification of knock-in efficacy

To determine the efficacy of knock-ins, samples were fixed at DIV 14, and stained with primary and secondary antibodies as described above. Coverslips were examined with epifluorescence on a Nikon Eclipse 80i upright microscope Plan Fluor 20× air (N.A. 0.75) or Plan Fluor 40× oil (N.A. 1.30) objective, CoolLED pE-300^white^ illumination and Chroma ET-GFP/mCherry (59 022) filter set. Coverslips were scanned top to bottom and fluorescent cells were scored manually based on staining pattern in one of four categories. For instance, for Cre_OFF_ β3-tubulin GFP/Cre_ON_ Halo-β-actin, the categories were (1) GFP correct (GFP staining pattern corresponds with β3-tubulin expression); (2) Halo correct (Halo staining pattern corresponds with β-actin pattern); (3) double correct (both GFP and Halo have the correct staining pattern in the same cell); (4) incorrect (GFP staining pattern corresponds with β-actin and/or Halo corresponds with β3-tubulin). Results were obtained from three independent cultures with one coverslip per culture, unless stated otherwise.

### Confocal microscopy

Confocal images were acquired with a Zeiss LSM 700, using a EC Plan-Neofluar 40× oil (N.A.1.30) or Plan-Apochromat 63× oil (N.A. 1.40) objective and 488-, 555-, and 633-nm laser excitation lines. Images were acquired as z-stacks containing planes at 0.5-μm interval, with 0.1-μm pixel size and 2× pixel averaging. All images are displayed as maximum intensity projections. Images in Extended Data Figure 1-2 were acquired as tile scans, and stitched using Zeiss Zen Black 2.3 SP1 software.

### Spinning disk microscopy with fluorescence recovery after photobleaching (FRAP)

Neurons were transfected at DIV 2 as described above with pOC2 Dlg4-Halo-FKBP (33 fmol), pOC1 Gria1-GFP-FRB (33 fmol), pCAG ER^T2^-Cre-ER^T2^ (2 fmol) and pCAG FLAG-Cas9 (90 fmol). At DIV 7, 4OH-tamoxifen (100 nm) was added to the neurons. At DIV 21, just before imaging, neurons were live-labeled with Halo-ligand JF549 (diluted 1:1000 in conditioned medium) for 15 min and washed in conditioned medium for 10 min before mounting.

Imaging was performed on a spinning disk confocal system (CSU-X1-A1; Yokogawa) mounted on a Nikon Eclipse Ti microscope (Nikon) with Plan Apo VC 100 × 1.40 NA oil objective (Nikon) with excitation from Cobolt Calyspso (491 nm) And Cobolt Jive (561 nm), and emission filters (Chroma). The microscope was equipped with a motorized XYZ stage (ASI; MS-2000), Perfect Focus System (Nikon), Prime BSI sCMOS camera (Photometrics), and was controlled by MetaMorph software (Molecular Devices). Neurons were mounted in a Ludin-chamber (Life Imaging Services) with 450 μl extracellular buffer (in mm: 140 NaCl, 3 KCl, 10 HEPES, 2.7 CaCl_2_, 2 MgCl_2_, and 10 D-glucose. pH 7.35) and maintained in a closed incubation chamber (Tokai hit: INUBG2E-ZILCS) at 37°C.

Double knock-in neurons were imaged for 15 min, acquiring a Z-stack of three planes (0.5-μm interval) every 5 min. Hereafter, preselected regions of interest (ROIs; 1.3 μm in diameter) on spines were bleached using the ILas2 system (Roper Scientific). After bleaching, the neurons were imaged every 5 min for a total of 30 min. Following the first acquisition, cells were incubated in 1 μm rapalog for 20 min. Next, a different part of the same neuron was selected for a second (after rapalog) round of FRAP imaging. As a control, Cre_ON_ GluA1-GFP-FRB-positive neurons were imaged following the same protocol, but without the addition of rapalog.

Acquisitions were corrected for drift and a maximum projection of the Z-stack was used for analysis. For each ROI and time point, mean intensities were measured and corrected for background and bleaching. Mean intensities were normalized to 1 using the averaged intensities of the frames before bleaching, and normalized to 0 based on the intensity from the first frame after bleaching. The mobile fraction of GluA1-GFP-FRB was calculated by averaging the normalized intensity of the last four frames for each ROI. Analysis was performed using FIJI and Excel.

Synapse enrichment before and after rapalog was calculated as the ratio between synapse and dendritic shaft intensity using 10 ROIs (250-nm diameter) each. For this analysis, we used the images from time point −15 min.

### Dual-color single-molecule localization microscopy (SMLM)

Neurons were transfected at DIV 1 as described above with pOC2 PSD95-GFP (7.9 fmol), pOC1 GluA1-HA (44 fmol), pCAG ERT2-Cre-ERT2 (2 fmol) and pCAG FLAG-Cas9 (90 fmol); 100 nm 4OH-tamoxifen was added at DIV 7. At DIV 23, cultures were fixed and stained with primary and secondary antibodies as described above. PSD95-GFP and GluA1-HA were labeled with Alexa647and CF568-conjugated secondary antibodies, respectively.

SMLM was performed on the Nanoimager S from ONI (Oxford Nanoimaging Ltd.), fitted with a 100 × 1.4 NA oil-immersion objective, four laser lines (405, 561, and 640 nm), an XYZ closed-loop piezo stage and a sCMOS camera (ORCA Flash 4, Hamamatsu). Integrated filters are used to split far-red emission onto the right side of the camera and blue-green-red emission spectra on the left side, enabling simultaneous dual-color imaging. The imaging chamber was temperature-controlled at 30°C to prevent fluctuations in temperature during the time course of an experiment that might affect the alignment of the channels. Channel alignment was performed before each imaging session using 100 nm TetraSpeck beads (T-7279, Invitrogen) and the ONI software aiming for an alignment error of standard deviation <7 nm as measured from 2000 points total across a maximum of 20 fields of view. Imaging was performed in near-TIRF using a motorized mirror. During acquisition, neurons were kept in a STORM buffer (pH 8.0) containing 50 mm Tris, 10 mm NaCl, 10% w/v D-glucose, 5 mm MEA, 700 μg/ml glucose oxidase, and 40 μg/ml catalase. For each double knock-in neuron, 20,000 frames were acquired at 50 Hz. NimOS software from ONI was used for detection of single molecule localization events. Resulting localization tables were drift-corrected using Detection of Molecules (DoM) plugin v.1.2.1 for ImageJ (https://github.com/ekatrukha/DoM_Utrecht). dSTORM reconstruction was made using DoM with pixel size of 12 × 12 nm. Analysis was continued in MATLAB (2021a).

Localizations were filtered out if localization precision was >30 nm for GluA1 and >25 nm for PSD95, or photon count was <300 or >30,000 photons. Consecutive localizations in a radius of 60 nm were removed. If consecutive localizations persisted for >10 frames, the initial localization was also removed. ROIs outlining the synapse were defined based on the full-width half maximum (FWHM) using a widefield image of PSD95-GFP taken before dSTORM acquisition. Synapses were only analyzed further if they contained >800 localizations for PSD95 and >400 localizations for GluA1, and if they were >0.02 or <0.3 μm^2^ in size. For each localization in a given synapse, the local density was calculated as the number of localizations within a radius of 5× the mean nearest neighbor distance (MNND; MacGillavry et al., 2013). Localizations were deemed part of a nanodomain if its local density was >40. Nanodomains were isolated using the MATLAB functions *linkage* and *cluster*. Subsequently, nanodomains were subclustered if they contained multiple local density peaks that were >80% of the maximum local density, further than 80 nm apart and separated by a local minimum of <30% of the maximum local density. The nanodomain boundary was constructed using Voronoi diagrams circumventing the localizations. Nanodomains with <5% of the localizations in a synapse or a diameter of < 30 nm were excluded. The center of the postsynaptic density (PSD) was calculated using the function centroid() and based on the PSD95 cluster inside the synapse, identified using DBSCAN (Ester et al., 1996). Nanodomain distance between PSD95 and GluA1 was calculated for each nanodomain as the center-to-center distance to its closest nanodomain in the other channel. Co-localization analysis of PSD95 and GluA1 was performed as described previously (Willems and MacGillavry, 2022). As a first step in determining the co-localization between two clusters, the local density is determined for each localization in both channels. The MNND is determined within the PSD using the MATLAB function *knnsearch*. Next, for each localization, the local density (*LD*) is defined as the number of localizations within a radius defined by the effective resolution making use of the MATLAB function *rangesearch*. For each channel, the *LD* values are averaged together to obtain *LD_A_* and *LD_B_.* Effective resolution was calculated as (Gould et al., 2009):

$$effective\;resolution=\sqrt{MNND^{2}+\varepsilon^{2}},$$

where ε is the localization error.

The co-localization index is determined as the number of localizations of channel B (*N*) within a radius (*d*) around each localization in channel A (*Ai*) normalized to the mean *LD* of the localizations in channel B ( ${\bar{LD}}_{B}$), with *d* being the effective resolution of the localizations in channel B:

$$CI_{i}^{A}\left(d\right)=\frac{N_{Ai}^{B}(d)}{{\bar{LD}}_{B}}.$$

Thus, for channel B, the co-localization index values are calculated as:

$$CI_{i}^{B}\left(d\right)=\frac{N_{Bi}^{A}(d)}{{\bar{LD}}_{A}}.$$

The co-localization indices calculated for each localization individually are used to plot a co-localization map and averaged to obtain a mean co-localization index per synapse for both channels.

### Data representation and statistics

All experiment were performed in at least three independent cultures. Data are shown as average values, error bars represent standard error of the mean. When comparing two experimental groups, an unpaired *t* test was used, except for the FRAP analysis, which was analyzed with a paired *t* test. If groups were not normally distributed, the nonparametric Mann–Whitney test was used. Cell counting experiments were analyzed with a one-way or two-way ANOVA, and for results with *p* < 0.05, a *post hoc* test with Tukey–Kramer correction was performed to test for differences between individual groups. Table 5 provides an overview of all statistical tests used per figure.

**Table 5:** Statistical table

Figure	Description	Data structure	Type of test	Power (statistic)
Fig. 3*A*	β3-tubulin (Cre_OFF_ DNA)	Normal distribution	Two-way ANOVA	*F*_(3,24)_ = 5.3
Fig. 3*A*	β-actin (Cre_OFF_ DNA)	Normal distribution	Two-way ANOVA	*F*_(3,24)_ = 4.6
Fig. 3*A*	Double (Cre_OFF_ DNA)	Normal distribution	Two-way ANOVA	*F*_(3,24)_ = 7.6
Fig. 3*A*	Incorrect (Cre_OFF_ DNA)	Normal distribution	Two-way ANOVA	*F*_(3,24)_ = 4.2
Fig. 3*B*	β3-tubulin (DNA)	Normal distribution	ANOVA	*F*_(7,16)_ = 3.9
Fig. 3*B*	β-actin (DNA)	Normal distribution	ANOVA	*F*_(7,16)_ = 3.4
Fig. 3B	Double (DNA)	Normal distribution	ANOVA	*F*_(7,16)_ = 2.3
Fig. 3*B*	Incorrect (DNA)	Normal distribution	ANOVA	*F*_(7,16)_ = 0
Fig. 3*E*	Donor copy number	Normal distribution	Linear regression	*F*_(1,10)_ = 7.2
Fig. 4*B*	β3-tubulin (4OH-tamoxifen)	Normal distribution	Two-way ANOVA	*F*_(3,24)_ = 0.49
Fig. 4*B*	β-actin (4OH-tamoxifen)	Normal distribution	Two-way ANOVA	*F*_(3,24)_ = 27.6
Fig. 4*B*	Double (4OH-tamoxifen)	Normal distribution	Two-way ANOVA	*F*_(3,24)_ = 14.4
Fig. 4*B*	Incorrect (4OH-tamoxifen)	Normal distribution	Two-way ANOVA	*F*_(3,24)_ = 3.1
Fig. 4*C*	Double (lenti vs tamoxifen)	Normal distribution	Unpaired *t* test	*t*_(13)_ = 1.8
Fig. 5*D*	GluA1 (DNA)	Normal distribution	Kruskal–Wallis	Statistic = 9.2
Fig. 5*D*	Ca_v_2.3 (DNA)	Normal distribution	Kruskal–Wallis	Statistic = 1.6
Fig. 5*D*	Double (DNA)	Normal distribution	Kruskal–Wallis	Statistic = 5.7
Fig. 5*D*	Incorrect (DNA)	Normal distribution	Kruskal–Wallis	Statistic = 3.0
Fig. 5*E*	Time (DIV)	Normal distribution	Two-way ANOVA	*F*_(3,35)_ = 12.5
Fig. 5*E*	Knock-in	Normal distribution	Two-way ANOVA	*F*_(2,35)_ = 13.3
Fig. 5*E*	Interaction	Normal distribution	Two-way ANOVA	*F*_(6,35)_ = 3.2
Fig. 5*G*	GluA1	Normal distribution	ANOVA	*F*_(2,6)_ = 0.099
Fig. 5*G*	Ca_v_2.3	Normal distribution	ANOVA	*F*_(2,6)_ = 2.4
Fig. 5*G*	Double	Normal distribution	ANOVA	*F*_(2,6)_ = 0.56
Fig. 5*G*	Incorrect	Normal distribution	ANOVA	*F*_(2,6)_ = 5.7
Fig. 6*C*	Nanodomain-PSD distance	Nonuniform	Mann–Whitney	Statistic = 426143
Fig. 6*D*	PSD size vs nanodomain distance	Nonuniform	Linear regression	*F*_(1,642)_ = 13.8
Fig. 6*F*	PSD size vs co-localization	Nonuniform	Linear regression	*F*_(1,654)_ = 155.7
Fig. 6*G*	PSD95 (nanodomain enrichment)	Normal distribution	One-sample *t* test	*t*_(11)_ = 8.1
Fig. 6*G*	GluA1 (nanodomain enrichment)	Normal distribution	One-sample *t* test	*t*_(11)_ = 11.6
Fig. 7*F*	Mobile GluA1 before/after	Normal distribution	Paired *t* test	*t*_(5)_ = 3.4
Fig. 7*G*	GluA1 synaptic enrichment	Normal distribution	Paired *t* test	*t*_(5)_ = 1.4
Extended Data Fig. 1-1*D*	β3-tubulin (2A vs lenti)	Normal distribution	Unpaired *t* test	*t*_(13)_ = 0.69
Extended Data Fig. 1-1*D*	β-actin (2A vs lenti)	Normal distribution	Unpaired *t* test	*t*_(13)_ = 1.1
Extended Data Fig. 1-1*D*	Double (2A vs lenti)	Normal distribution	Unpaired *t* test	*t*_(13)_ = 0.64
Extended Data Fig. 1-1*D*	Incorrect (2A vs lenti)	Normal distribution	Unpaired *t* test	*t*_(13)_ = 2.7
Extended Data Fig. 1-1*E*	β3-tubulin (2A vs lenti)	Normal distribution	Unpaired *t* test	*t*_(44)_ = 4.5
Extended Data Fig. 1-1*E*	β-actin (2A vs lenti)	Normal distribution	Unpaired *t* test	*t*_(30)_ = 0.29
Extended Data Fig. 3-1*A*	GluA1 (Cre_OFF_ DNA)	Normal distribution	Two-way ANOVA	*F*_(3,24)_ = 2.2
Extended Data Fig. 3-1*A*	PSD95 (Cre_OFF_ DNA)	Normal distribution	Two-way ANOVA	*F*_(3,24)_ = 2.5
Extended Data Fig. 3-1*A*	Double (Cre_OFF_ DNA)	Normal distribution	Two-way ANOVA	*F*_(3,24)_ = 1.3
Extended Data Fig. 3-1*A*	Incorrect (Cre_OFF_ DNA)	Normal distribution	Two-way ANOVA	*F*_(3,24)_ = 0.33
Extended Data Fig. 3-1*C*	β3-tubulin (plasmid)	Normal distribution	Two-way ANOVA	*F*_(2,24)_ = 18.6
Extended Data Fig. 3-1*C*	β-actin (plasmid)	Normal distribution	Two-way ANOVA	*F*_(2,24)_ = 0.72
Extended Data Fig. 3-1*C*	Double (plasmid)	Normal distribution	Two-way ANOVA	*F*_(2,24)_ = 11.4
Extended Data Fig. 3-1*C*	Incorrect (plasmid)	Normal distribution	Two-way ANOVA	*F*_(2,24)_ = 0
Extended Data Fig. 4-1*B*	PSD95 (4OH-tamoxifen)	Normal distribution	Two-way ANOVA	*F*_(3,24)_ = 0.16
Extended Data Fig. 4-1*B*	GluA1 (4OH-tamoxifen)	Normal distribution	Two-way ANOVA	*F*_(3,24)_ = 4.8
Extended Data Fig. 4-1*B*	Double (4OH-tamoxifen)	Normal distribution	Two-way ANOVA	*F*_(3,24)_ = 1.7
Extended Data Fig. 4-1*B*	Incorrect (4OH-tamoxifen)	Normal distribution	Two-way ANOVA	*F*_(3,24)_ = 1
Extended Data Fig. 6-1*A*	Number of nanodomains	Normal distribution	Unpaired *t* test	*t*_(22)_ = 10.0
Extended Data Fig. 6-1*A*	Nanodomain diameter	Normal distribution	Unpaired *t* test	*t*_(22)_ = 7.9
Extended Data Fig. 7-1*D*	Mobile GluA1 (no rapalog)	Normal distribution	Paired *t* test	*t*_(5)_ = 0.18

Overview of all statistical tests that were performed, including figure number, description, data structure, the type of test and the power (statistic).

## Results

### CAKE creates double knock-ins in neurons

We reasoned that accurate multiplex knock-ins in neurons could be achieved by separating genome editing events in time using a Cre-dependent conditional activation mechanism (Fig. 1*A*,*B*). In a previous study we achieved this by fusing GFP-2A-Cre to the first gene to activate a second knock-in construct with a Cre-dependent single gRNA (Willems et al., 2020; Extended Data Fig. 1-1*A*). This mechanism successfully yielded double knock-in cells for a variety of gene combinations, illustrating the potential of sequential gene editing (Willems et al., 2020). However, appreciable cross talk between the knock-ins still occurred, suggesting we had insufficient control over the delay between genome editing events (Extended Data Fig. 1-1*C*).

Here, we introduce two major improvements of this CAKE strategy (Fig. 1*C*). First, to obtain full control over the switch from the first to the second gRNA, we separately introduced Cre expression. We either used lentiviral infection of a Cre-expressing vector or lipofection of a 4OH-tamoxifen-inducible Cre-expressing construct. Second, to reduce cross talk, we redesigned the first knock-in vector such that gRNA expression of the first vector is switched off by Cre, effectively limiting further editing of the first targeted locus (also see Chylinski et al., 2019). We refer to the first knock-in vector as Cre_OFF_ (gRNA expression is switched off by Cre), and the second knock-in vector as Cre_ON_ (gRNA expression switched on by Cre; see Fig. 1*B*). This sequential knock-in strategy yielded a mosaic of fluorescent cells, with cells positive for the Cre_OFF_ or Cre_ON_ knock-ins (Extended Data Fig. 1-2) and a fraction of double knock-in cells that are positive for both the Cre_ON_ and Cre_OFF_ knock-ins (Fig. 1*D*).

We first compared the updated CAKE strategy with our previous GFP-2A-Cre based method (Willems et al., 2020), using knock-in constructs for β3-tubulin and β-actin, delivered to cultured rat hippocampal neurons with lipofectamine (Extended Data Fig. 1-1). Since the distribution patterns of β3-tubulin and β-actin in neurons are well-known to be segregated in different subcellular compartments, this allowed us to easily quantify knock-in efficacy and accuracy. We systematically counted all fluorescent cells per coverslip, scoring them as a correct knock-in cell for β3-tubulin-GFP or Halo-β-actin; a double knock-in cell, or, if donor cross talk had occurred, as an incorrect knock-in cell. Strikingly, while both methods lead to a similar number of single and double knock-in cells, the updated strategy nearly completely abolished cross talk between knock-ins (Extended Data Fig. 1-1*D*). Furthermore, we noted that for some genes GFP-2A-Cre fusion resulted in reduced expression levels, probably because of the increase in mRNA length (Willems et al., 2020). Importantly, no such effect was found for the improved CAKE method (Extended Data Fig. 1-1*E*). Thus, CAKE faithfully created double knock-ins in cultured hippocampal neurons.

### CAKE can be applied to multiple gene combinations

**Figure 2.**
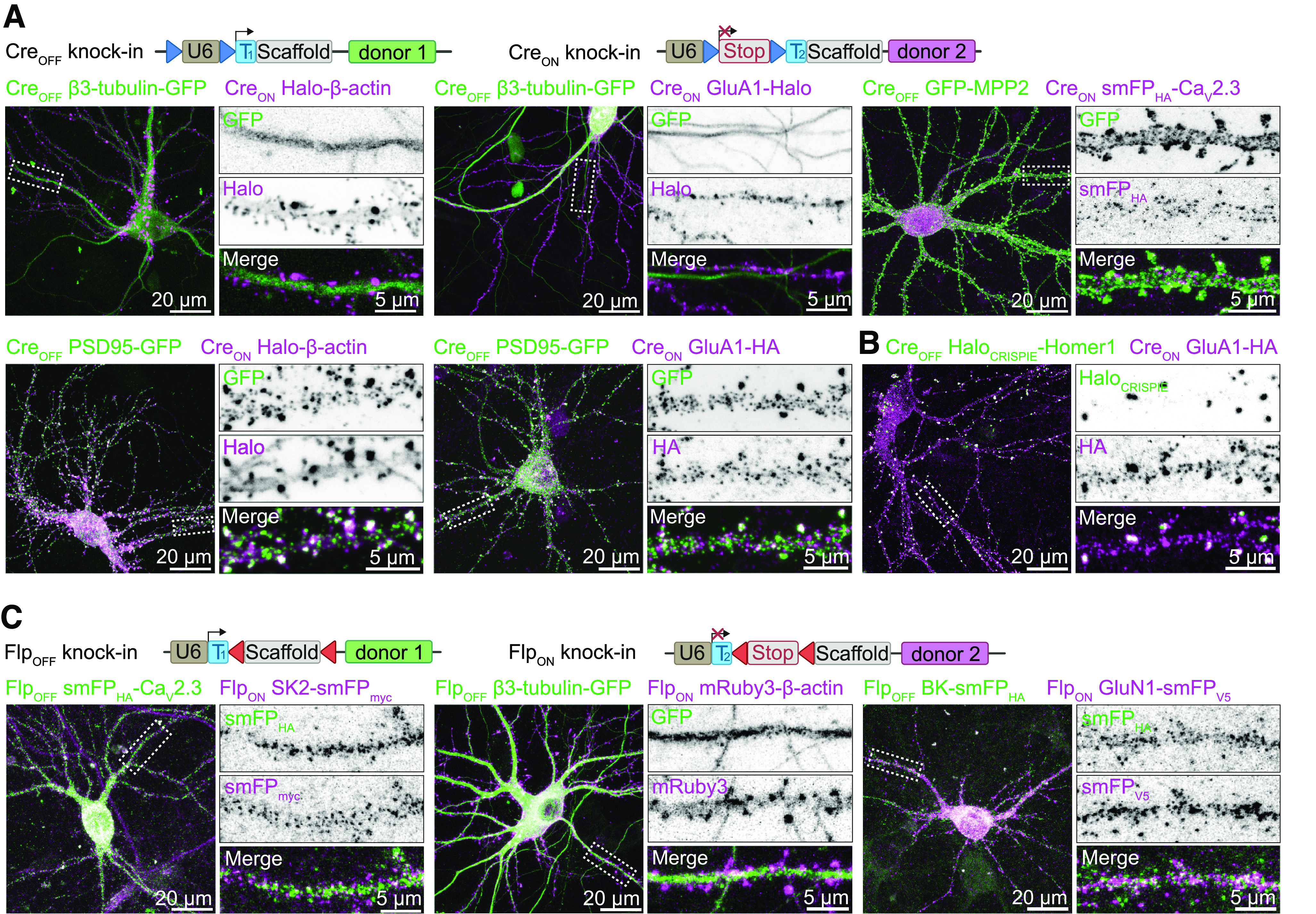
CAKE generates double knock-ins for a variety of genes. ***A***, Top, Overview of Cre_OFF_ and Cre_ON_ knock-in constructs. Bottom, example confocal microscopy images of CAKE knock-in cells controlled by Cre-recombinase. For Cre_OFF_ β3-tubulin-GFP/Cre_ON_ GluA1-Halo, lenti GFP-Cre was used, which labels infected nuclei. The remaining examples were obtained with lenti-Cre without GFP, or with ER^T2^-Cre-ER^T2^. Lentivirus or 100 nm 4OH-tamoxifen was added at DIV 7. Cells were fixed at DIV 14. ***B***, Example confocal image of Cre_OFF_ Halo_CRISPIE_-Homer1 and Cre_ON_ GluA1-HA double knock-in cells. Halo_CRISPIE_ donor DNA is inserted into intron 1 of Homer1 as a novel exon (see Extended Data Fig. 2-1; Zhong et al., 2021). Double knock-in was obtained using ER^T2^-Cre-ER^T2^ and 100 nm 4OH-tamoxifen was added at DIV 7. Cells were fixed at DIV 14. ***C***, Top, overview of Flp_OFF_ and Flp_ON_ knock-in constructs. Bottom, example confocal microscopy images of CAKE knock-ins controlled by lenti-Flp^O^-recombinase, added on DIV 7. Cells were fixed at DIV 14.

To test whether we could generalize the application of CAKE to other gene pairs, we generated a set of Cre_OFF_ and Cre_ON_ knock-in vectors targeting a diversity of neuronal proteins. These knock-ins include synaptic scaffolding proteins (PSD95, Homer1, and MPP2) and neurotransmitter receptor subunits (GluA1, GluN1). We also added voltage-gated and Ca^2+^-gated ion channels (Ca_V_2.3, SK2, BK), where the limited availability of antibodies has hampered (co-)localization analysis in neurons. Similar to β3-tubulin and β-actin knock-in vectors, these CAKE combinations yielded mosaic fluorescent labeling in cultured neurons. Importantly, we identified multiple double knock-in cells for many gene combinations (Fig. 2*A*). This illustrates the potential for CAKE to study the spatiotemporal co-expression of a wide range of proteins.

10.1523/ENEURO.0056-22.2022.f1-1 Extended Data Figure 1-1 Improved CAKE mechanism reduces cross talk between knock-ins. ***A***, Overview of original CAKE knock-in mechanism introduced by Willems et al. (2020), referred to as 2A-Cre throughout this Figure. The constitutively active knock-in labels β3-tubulin with GFP-2A-Cre. Cre expression from this allele then activates gRNA expression for the Cre_ON_ Halo-β-actin knock-in. ***B***, Overview of improved CAKE constructs introduced in this study (see main text) which will be referred to as Lenti-Cre throughout this figure. Lentivirus encoding for Cre is added at DIV 7, which switches off gRNA expression for Cre_OFF_, and switches on gRNA expression for Cre_ON_. ***C***, Example confocal images of CAKE double knock-ins using the 2A-Cre or Lenti-Cre mechanism. ***D***, Average number of single and double knock-in cells per coverslip for 2A-Cre and Lenti-Cre CAKE. 2A-Cre *n* = 8 coverslips, lenti-Cre *n* = 7 coverslips, *N* = 4 independent cultures. β3-tubulin-GFP *p* = 0.50, Halo-β-actin *p* = 0.29, double knock-in *p* = 0.53, incorrect knock-in *p = 0.019, unpaired *t* test. ***E***, Average fluorescence intensity in proximal dendrites of single knock-ins, normalized per culture. For β3-tubulin-GFP: 2A-Cre *n* = 21 cells, lenti-Cre *n* = 25 cells, *N* = 3 independent cultures. For Halo-β-actin: 2A-Cre *n* = 18 cells, lenti-Cre *n* = 14 cells, *N* = 3 independent cultures. β3-tubulin-GFP *p = 0.001, Halo-β-actin *p* = 0.29, unpaired *t* test. ***F***, Example confocal image of an incorrect double knock-in. Fluorescence signal of both GFP and Halo are consistent with β3-tubulin distribution, suggesting that the Halo donor was inserted in Tubb3. Download Figure 1-1, EPS file.

10.1523/ENEURO.0056-22.2022.f1-2 Extended Data Figure 1-2 Low-magnification images of CAKE knock-in cultures. ***A***, Low-magnification images of DIV 14 culture transfected with Cre_OFF_ β3-tubulin-GFP and Cre_ON_ Halo-β-actin knock-in vectors. Control is unstimulated, +Cre condition was infected with 20-μl lenti-Cre at DIV 7. Notice that CAKE leads to a mosaic of single and double knock-ins (indicated with *). Fluorescent neurons are surrounded by untransfected cells. ***B***, As in ***A***, for Cre_OFF_ PSD95-GFP and Cre_ON_ GluA1-Halo. Download Figure 1-2, EPS file.

**Figure 3.**
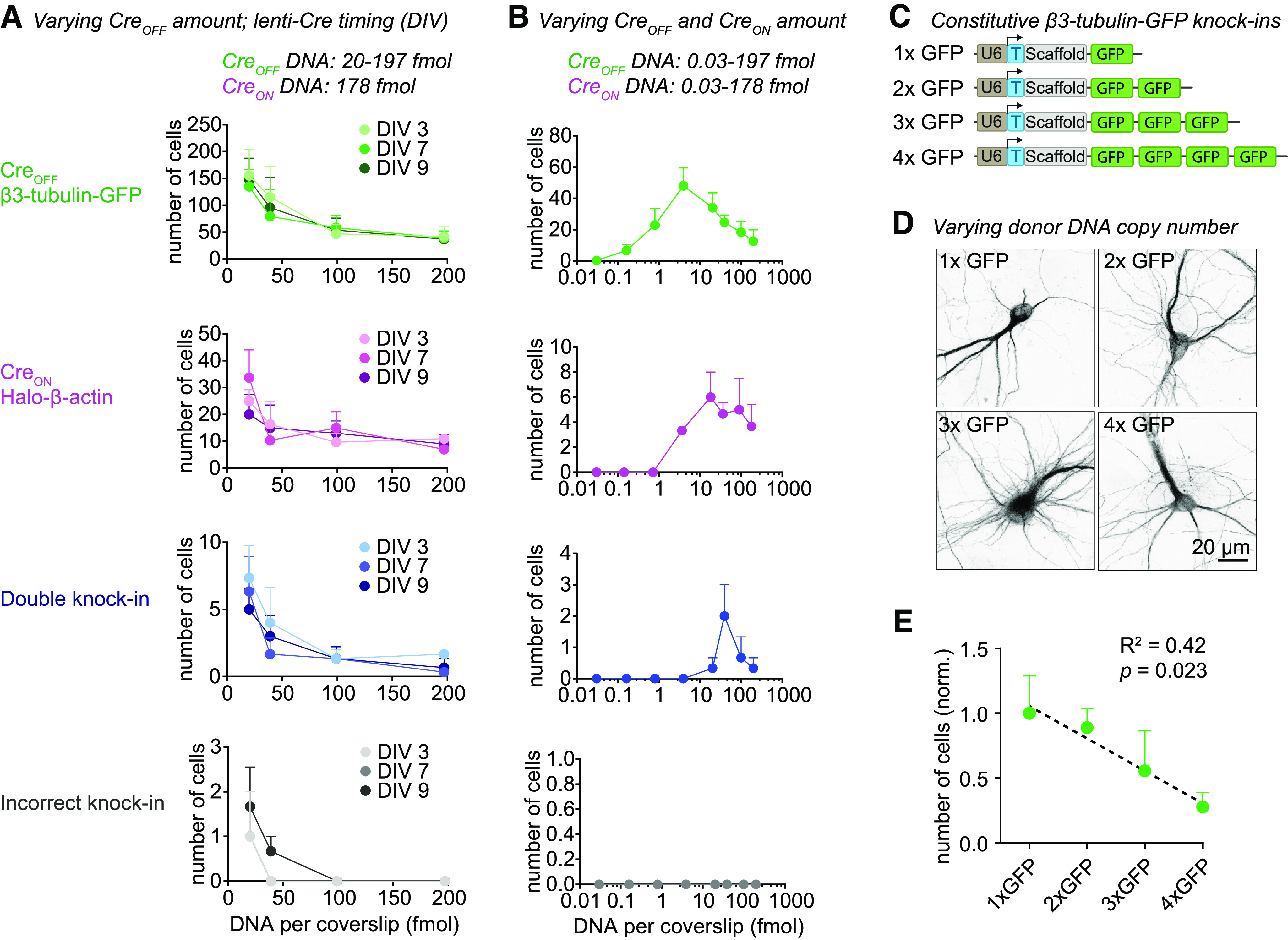
Donor DNA amount controls knock-in efficacy. ***A***, Number of fluorescent cells per coverslip for each knock-in, as a function of Cre_OFF_ β3-Tubulin-GFP vector amount; 20 μl lenti-Cre was added at DIV 3, 7, or 9. *n* = 3 coverslips, *N* = 3 independent cultures. This experiment was repeated for Cre_OFF_ GluA1-GFP and Cre_ON_ PSD95-Halo (see Extended Data Fig. 3-1*A*). ***B***, Number of fluorescent cells per coverslip for each knock-in. Both the amount of Cre_OFF_ and Cre_ON_ vector were varied; 20 μl lenti-Cre was added at DIV 7. *n* = 3 coverslips, *N* = 3 independent cultures. ***C***, pORANGE β3-tubulin knock-in constructs used to titrate the amount of donor DNA. Each GFP donor has its own PAM and target sequence (data not shown), and thus every GFP donor can be cleaved independently from the vector. ***D***, Example confocal images of β3-tubulin-GFP knock-in cells using one to four GFP donors per vector. ***E***, Number of fluorescent cells as a function of number of GFP donors. Data were normalized to average number of knock-ins in the 1xGFP condition. *R*^2^ = 0.42, *p* = 0.023, model linear regression (dotted black line). *n* = 3 coverslips, *N* = 3 independent cultures.

10.1523/ENEURO.0056-22.2022.f1-3 Extended Data Figure 1-3 Overview and cloning guide for CAKE template vector library. ***A***, Overview of pORANGE (Willems et al., 2020) and the CAKE template vectors introduced in this study. All vectors contain a multiple cloning site for addition of the donor DNA. ***B***, Cloning guide to create CAKE knock-in constructs. The example shows a step-by-step protocol for pOC2 Cre_OFF_ knock-in vectors, but identical cloning steps apply to all pOC vectors shown in ***A***. #1 shows the empty pOC2 template vector. After digestion with BbsI, a primer pair encoding for the gRNA target sequence is added to obtain the intermediate construct (#2). The donor DNA, which always contains a protospacer adjacent motif (PAM) and target sequence (Suzuki et al., 2016) is obtained with standard PCR techniques. Willems et al. (2020), their Figure S1, contains a detailed description on design of the donor DNA. The donor is cloned using HindIII and MluI restriction sites to obtain the final pOC2 knock-in construct (#3). Most of the knock-in constructs used in this study contain BmtI and AfeI restriction sites around the fluorophore, for universal exchange of donors between knock-in vectors, without the need of PCR (shown in #4, optional). The entire knock-in cassette, containing U6-driven gRNA expression, Lox551 and/or Frt sites and the donor DNA, can be removed from pOC vectors using PscI and MluI restriction sites. A complete description of Cas9 target sequence selection, knock-in design and cloning can be found in Extended Data Figure 1-4. Download Figure 1-3, EPS file.

**Figure 4.**
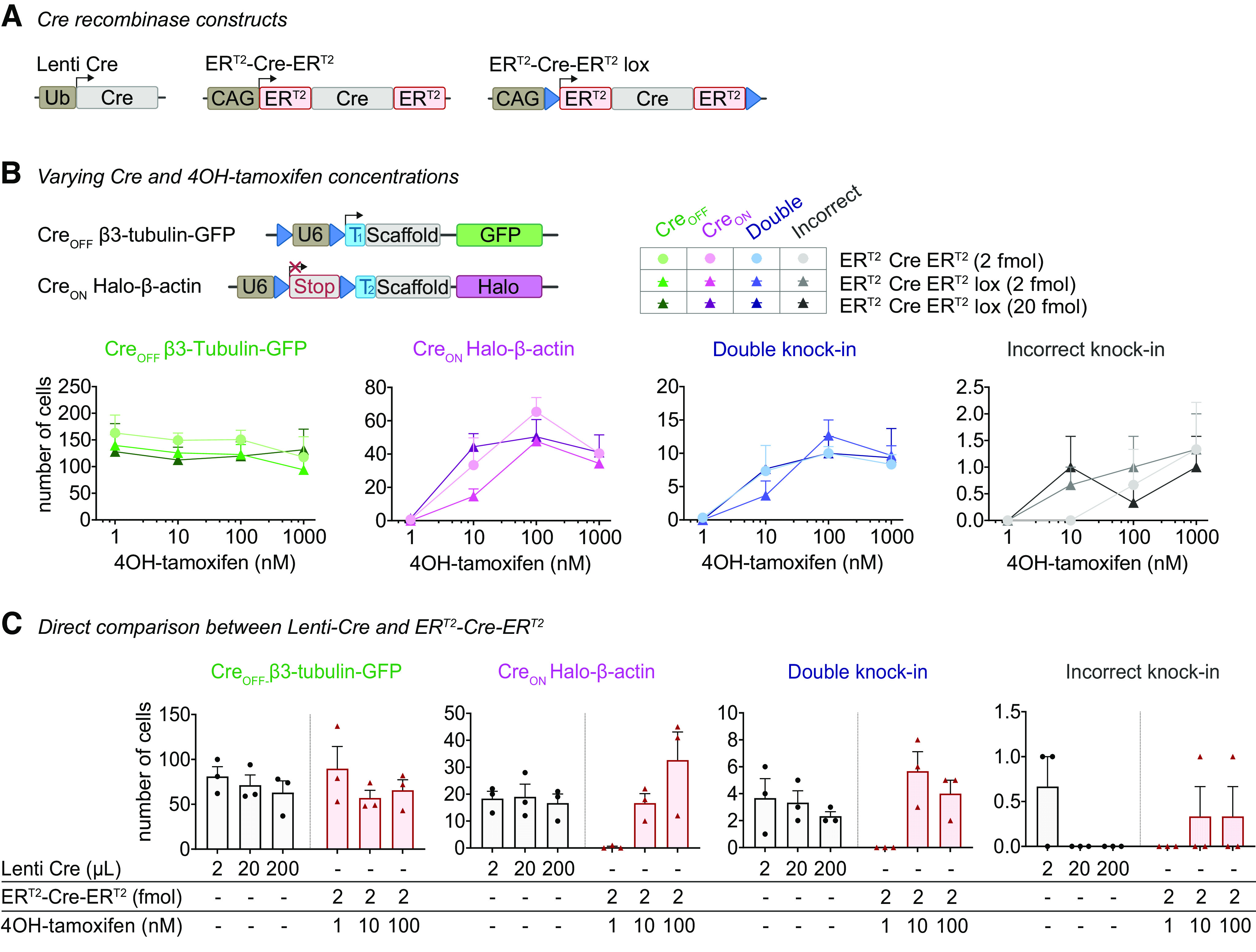
Comparison of methods to deliver and activate Cre-recombinase. ***A***, Overview of Cre-recombinase constructs. ***B***, Number of fluorescent knock-in cells per coverslip using ER^T2^-Cre-ER^T2^ constructs. 4OH-tamoxifen was added at the indicated concentration at DIV 7. *n* = 3 coverslips, *N* = 3 independent cultures. 71% of knock-in cells was β3-tubulin-GFP-positive, 24% was Halo-β-actin-positive, and 5.0% was double positive. A similar experiment was performed using Cre_OFF_ PSD95-GFP and Cre_ON_ GluA1-HA (see Extended Data Fig. 4-1*B*). ***C***, Comparison of Lenti-Cre and ER^T2^-Cre-ER^T2^. Lenti-Cre and 4OH-tamoxifen were added at DIV 7. *n* = 3 coverslips, *N* = 3 independent cultures.

10.1523/ENEURO.0056-22.2022.f1-4 Extended Data Figure 1-4 Step-by-step guidelines for making ORANGE CAKE knock-ins, including genomic target sequence selection (***A***), design of primers for gRNA and donor DNA (***A***), and cloning into any CAKE template vector (***B***, ***C***). Download Figure 1-4, DOCX file.

10.1523/ENEURO.0056-22.2022.f2-1 Extended Data Figure 2-1 Overview of CRISPIE strategy. ***A***, Overview of DNA constructs used for the CRISPIE double knock-in. ***B***, Visual representation of the CRISPIE modality. Halo, flanked by the splicing acceptor and donor sites of exon 8 of Septin3, is inserted into the first intron of Homer1 following Cas9 cleavage. Halo then functions as exon1’ and will be integrated in the mRNA. Download Figure 2-1, EPS file.

### CAKE is compatible with multiple CRISPR-Cas9 knock-in strategies

Because CAKE is based on sequential gRNA expression, we reasoned that our method should be compatible with other recently developed CRISPR/Cas9 knock-in strategies (Schmid-Burgk et al., 2016; Gao et al., 2019; Fang et al., 2021; Zhong et al., 2021). To assess the flexibility of CAKE, we implemented the CRISPR-mediated insertion of exon (CRISPIE) approach, which introduces designer exons in intronic sequences to mitigate the effect of indel mutations (Zhong et al., 2021). We inserted an exon containing Halo into the first intron of *Homer1* by flanking the donor DNA with splicing acceptor and donor sites (Extended Data Fig. 2-1). The resulting Cre_OFF_ vector was successfully combined with a Cre_ON_ ORANGE vector to attain double knock-in cells (Fig. 2*B*). Thus, various NHEJ-based CRISPR-Cas9 methods can be adopted and combined with CAKE to create multiplex knock-ins.

### Controlling CAKE with Flp-recombinase

To extend the utility of CAKE, we created CAKE vectors that are controlled by Flp-recombinase (Flp_OFF_ and Flp_ON_; Extended Data Fig. 1-3; Fig. 2*B*). The Frt and stop codon sequence were contained within the gRNA, which was reported to have a higher efficacy compared with integration in the U6 promoter (Chylinski et al., 2019). The switch between Flp_OFF_ and Flp_ON_ gRNA expression was controlled using a lentivirus expressing Flp^O^ (lenti-Flp). Flp-controlled CAKE performed comparable to Cre-controlled CAKE, and resulted in single and double knock-in cells for a variety of gene combinations (Fig. 2*C*). Finally, we developed a Cre_ON_ Flp_OFF_ knock-in vector, enabling intersectional activation of gRNA expression (Extended Data Fig. 1-3*A*). Thus, CAKE can be performed with both Cre- and Flp-recombinase.

### Knock-in efficacy is modulated by donor DNA levels

Initially, the number of double knock-in cells per coverslip was too low for many applications (Extended Data Fig. 1-1*D*). Therefore, we next set out a series of experiments to increase the number of double knock-in cells per sample, using Cre_OFF_ β3-tubulin-GFP and Cre_ON_ Halo-β-actin knock-in vectors. In early experiments we noticed that vector amounts used in transfection affected knock-in efficacy. To thoroughly test this, we systematically varied the amount of the Cre_OFF_ knock-in vector in our transfection mixture between 20 and 197 fmol (which equals to 50–500 ng DNA) per coverslip, while keeping Cre_ON_ fixed at 178 fmol, and scored all fluorescent cells per coverslip at DIV 14. Strikingly, we found a strong inverse relationship between Cre_OFF_ β3-tubulin-GFP vector amount and the number of β3-tubulin-GFP-positive cells (Fig. 3*A*, *p *=* *0.006, two-way ANOVA). Furthermore, lower Cre_OFF_ amounts also increased the number of Halo-β-actin-positive cells, although we kept the amount of Cre_ON_ vector constant in all conditions (Fig. 3*A*, *p *=* *0.04. two-way ANOVA). This interplay suggests competition between the two knock-ins, which continues after the Cre-dependent switch has occurred. Together with an increase in single knock-in cells, we observed a strong increase in double knock-in cells to between 5 and 8 cells per coverslip at the lowest Cre_OFF_ β3-tubulin-GFP amount (Fig. 3*A*, *p *=* *0.001, two-way ANOVA). In that condition, 82% of knock-in cells was β3-tubulin-GFP-positive, 14% was Halo-β-actin-positive, and 3.4% was double positive. Crucially, the number of incorrect knock-in neurons (i.e., donor cross talk for one of the targeted genes) remained low (one to two cells per coverslip, <1% of all knock-in cells), although the absolute number slightly increased with lower DNA amount (*p *=* *0.02, two-way ANOVA). In the same experiment, we tested whether the timing of Cre infection (infection at DIV 3, 7, or 9) affects the number of knock-in neurons. In contrast to the strong effect of vector amount, the timing of Cre expression did not influence the number of single or double knock-in neurons (Fig. 3*A*, Cre_OFF_ *p *=* *0.90, Cre_ON_ *p *=* *0.88, double knock-in *p *=* *0.49, incorrect knock-in *p *=* *0.50, two-way ANOVA), suggesting that either knock-in efficacy for this gene combination is insensitive to Cre timing, or that the onset of lentiviral-mediated Cre expression is too slow to observe an effect (in the order of days; Hioki et al., 2007). Similar results were obtained for Cre_OFF_ GluA1-GFP and Cre_ON_ PSD95-Halo, although the sensitivity for DNA amount appeared to differ between individual knock-in constructs (Extended Data Fig. 3-1*A*). We then tested whether further reduction of vector amount enhanced knock-in efficacy, and varied the amount of both the Cre_OFF_ and Cre_ON_ vectors. Here, we found that the optimum for Cre_OFF_ β3-tubulin-GFP is around 3.9 fmol DNA per coverslip. Cre_ON_ Halo-β-actin performed best around 36 fmol per coverslip, and efficacy dropped steeply below 3.6 fmol (Fig. 3*B*). This is in line with our previous observation that increased amounts of Cre_OFF_ knock-in vector negatively affect the Cre_ON_ knock-in efficacy (Fig. 3*A*).

10.1523/ENEURO.0056-22.2022.f3-1 Extended Data Figure 3-1 Donor DNA amount controls the number of knock-ins. ***A***, Number of fluorescent cells per coverslip for each knock-in, as a function of Cre_OFF_ GluA1-GFP vector amount; 20-μl lenti-Cre was added at DIV 3, 7 or 9. *n* = 3 coverslips, *N* = 3 independent cultures. For incorrect knock-ins, only double-incorrect cells were scored. A total of 86% of knock-in cells was GluA1-GFP-positive, 14% was PSD95-Halo-positive, and 2.2% was double positive. ***B***, Overview of conditions and constructs. Cre_OFF_ β3-tubulin-GFP is the same vector as used in the main text (for instance, in Fig. 1). The Cre_OFF_ gRNA is the same vector, but without the GFP donor DNA. Minicircle donor encodes for the GFP donor only, with a single protospacer adjacent motif and target sequence (Suzuki et al., 2016). ***C***, Number of fluorescent cells per coverslip for each knock-in. ER^T2^-Cre-ER^T2^ was used for this experiment, and 100 nm 4OH-tamoxifen was added at DIV 7. *n* = 3 coverslips, *N* = 3 independent cultures. Download Figure 3-1, EPS file.

We hypothesized that the inverse relationship between DNA amount and knock-in efficacy is because of competition between donor DNAs. To test whether increasing donor DNA copies per cell decreases knock-in efficacy, we generated ORANGE β3-tubulin knock-in vectors with one, two, three, or four independent copies of the donor DNA (Fig. 3*C–E*). Using these vectors, we found a clear negative correlation between the number of donor DNA copies and the number of β3-tubulin-GFP-positive cells per coverslip (Fig. 3*E*, *p *=* *0.023, *R*^2^ = 0.42).

Finally, to separate effects of gRNA and donor DNA levels, we replaced the Cre_OFF_ β3-tubulin-GFP vector with a Cre_OFF_ gRNA vector and a minicircle GFP donor (Extended Data Fig. 3-1*B*). Previous studies found that minicircle donors often outperform large donor plasmids, likely because Cas9-mediated cleavage of the donor plasmid can lead to integration of the vector backbone (Schmid-Burgk et al., 2016; Suzuki et al., 2016; Danner et al., 2021). We found that, at high donor levels, minicircle donor DNA performed similar to ORANGE donor plasmids, but knock-in efficacy was reduced at lower minicircle levels (Extended Data Fig. 3-1*C*). The reason for this reduction is unclear, but we cannot exclude that at low amounts of minicircle DNA the transfection efficacy is reduced. Similar to knock-in vector-delivered donor, the high amounts of the (Cre_OFF_-activated) minicircle donor also decreased the efficacy of the Cre_ON_ Halo-β-actin knock-in (Extended Data Fig. 3-1*C*). Importantly, increasing Cre_OFF_ gRNA expression level had no effect on knock-in efficacy for both the Cre_OFF_ and Cre_ON_ knock-in. Thus, under our experimental conditions, a single vector containing both the gRNA and donor DNA leads to the highest knock-in efficacy. Taken together, we conclude that donor DNA levels modulate knock-in efficacy.

### Tamoxifen-inducible Cre controls Cre_ON_ knock-in efficacy

In the experiments described above, we consistently observed a lower efficacy of Cre_ON_ compared with the Cre_OFF_ knock-ins. To improve the efficacy of Cre_ON_ knock-ins, we switched to 4OH-tamoxifen inducible Cre (ER^T2^-Cre-ER^T2^), which ensures rapid onset of Cre activation (Matsuda and Cepko, 2007) and superior control over the timing of Cre activity, compared with lentiviral-mediated Cre expression. As found by others (Forni et al., 2006; Higashi et al., 2009), we observed that strong, sustained activation of ER^T2^-Cre-ER^T2^ appears toxic to neurons, which resulted in fewer single and double knock-in cells (Extended Data Fig. 4-1*B*). To prevent toxicity, we reduced the vector encoding for ER^T2^-Cre-ER^T2^ to 2 fmol per coverslip, and we developed a self-inactivating Cre, by flanking ER^T2^-Cre-ER^T2^ with LoxP sites (ER^T2^-Cre-ER^T2^ lox, adapted from Pfeifer et al., 2001; Silver and Livingston, 2001). Under these conditions, 4OH-tamoxifen induced a dose-dependent increase in Cre_ON_ and double knock-ins (Cre_ON_ *p *=* *0.0001; double knock-in *p *=* *0.0001, two-way ANOVA), without affecting the number of Cre_OFF_ knock-in cells (Fig. 4*B*, *p *=* *0.70, two-way ANOVA). A 10-fold increase in ER^T2^-Cre-ER^T2^ lox vector (to 20 fmol) increased the number of Cre_ON_ at low 4OH-tamoxifen concentrations (Fig. 4*B*, *p *=* *0.025, two-way ANOVA *post hoc* comparison), but did not further increase efficacy at 100 or 1000 nm 4-OH-tamoxifen. Overall, no statistical difference was observed between ER^T2^-Cre-ER^T2^ and ER^T2^-Cre-ER^T2^ lox conditions (Cre_OFF_ knock-ins *p *=* *0.44, Cre_ON_ knock-ins *p *=* *0.10, double knock-ins *p *=* *0.98, two-way ANOVA). The number of incorrect knock-ins remained low (∼1 per coverslip at 1000 nm 4OH-tamoxifen), and was also similar between the Cre conditions (Fig. 4*B*, *p *=* *0.80, two-way ANOVA). Thus, at low vector concentrations ER^T2^-Cre-ER^T2^ accurately controls gRNA expression, while minimizing cytotoxicity.

10.1523/ENEURO.0056-22.2022.f4-1 Extended Data Figure 4-1 Optimization of lenti-Cre and ER^T2^-Cre-ER^T2^. ***A***, Number of fluorescent cells per coverslip as a function of the amount of lenti-Cre used for infection. Experiments were performed with Cre_OFF_ β3-tubulin-GFP and Cre_ON_ Halo-β-actin. Lenti-Cre was added at DIV 7. *n* = 2 coverslips *N* = 2 independent cultures. 70% of knock-in cells was PDS95-GFP-positive, 30% was GluA1-HA-positive, and 6.9% was double positive. ***B***, Number of fluorescent cells per coverslip as a function of 4OH-tamoxifen concentration and amount of pCAG ER^T2^-Cre-ER^T2^ vector at transfection. At 2 fmol (10 ng) pCAG ER^T2^-Cre-ER^T2^, 4OH-tamoxifen results in a dose-dependent increase in the number of Cre_ON_ knock-ins. Higher vector load (10–20 fmol) does not lead to more knock-in cells but increases ER^T2^-Cre-ER^T2^ cytotoxicity at 100 nm 4OH-tamoxifen. *n* = 3 coverslips, *N* = 3 independent cultures. Download Figure 4-1, EPS file.

Finally, we directly compared lenti-Cre and ER^T2^-Cre-ER^T2^, and found that ER^T2^-Cre-ER^T2^ did not boost the number of double knock-in neurons significantly (Fig. 4*C*). Taken together, multiple methods for Cre delivery and activation can be used to control CAKE, without obvious differences in the number of knock-in neurons. All subsequent experiments are based on ER^T2^-Cre-ER^T2^, activated on DIV 7 with 100 nm 4OH-tamoxifen.

### Cross talk between knock-ins is dependent on timing of Cre activation

**Figure 5.**
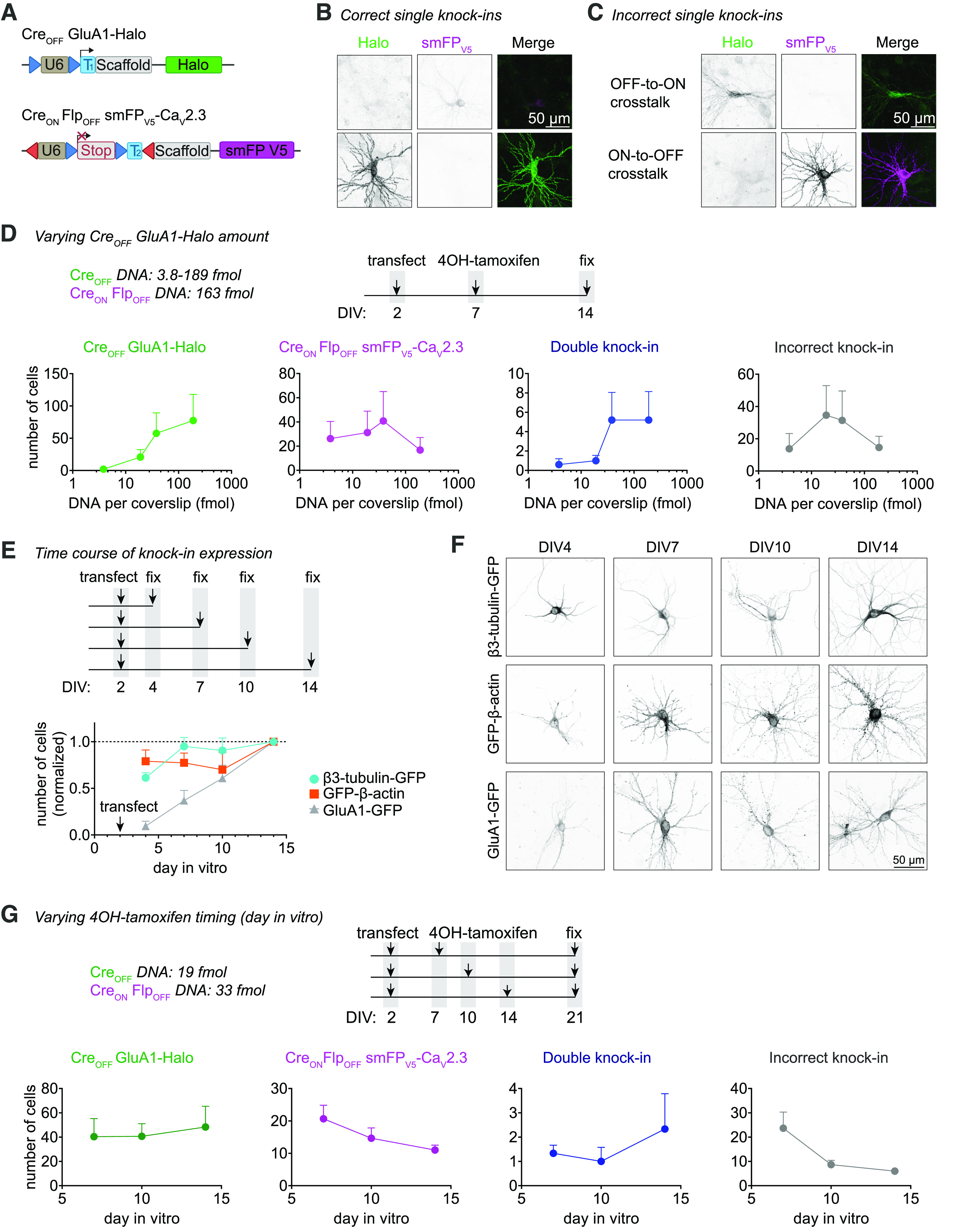
Delaying Cre activation reduces knock-in cross talk. ***A***, Overview of used DNA constructs. ***B***, ***C***, Example images of correct (***B***) and incorrect (***C***) knock-in neurons. Cells were incubated with 100 nm 4OH-tamoxifen at DIV 7, and fixed at DIV 14. Images in ***B***, ***C*** were taken with same acquisition settings to illustrate differences in expression level between Ca_V_2.3 (top) and GluA1 (bottom). ***D***, Top, experimental design. Bottom, number of fluorescent cells per coverslip for each knock-in, as a function of Cre_OFF_ GluA1-Halo vector amount. 100 nm 4OH-tamoxifen was added at DIV 7. Cells were fixed at DIV 14. *n* = 5 coverslips, *N* = 5 independent cultures. ***E***, Top, experimental design. Experiment was performed without Cre or 4OH-tamoxifen. Bottom, number of fluorescent cells per coverslip at different timepoints, normalized to the number of cells at DIV 14. *n* = 4 coverslips, *N* = 4 independent cultures. ***F***, Example confocal microscopy images of cells fixed at different DIVs from ***E***. Image acquisition settings were kept identical per knock-in. ***G***, Top, experimental design. Bottom, number of fluorescent cells per coverslip for each knock-in, as a function of the DIV at which 100 nm 4OH-tamoxifen was added. *n* = 3 coverslips, *N* = 3 independent cultures.

While testing several CAKE combinations, we noticed that one particular combination, using Cre_OFF_ GluA1-Halo and Cre_ON_ Flp_OFF_ smFP_V5_-Ca_V_2.3, showed an unusually high rate of donor cross talk (Fig. 5*A–C*). Specifically, ∼90% of these incorrect knock-ins were smFP_V5_-positive in a staining pattern expected for GluA1 (ON-to-OFF cross talk). To understand why the CAKE mechanism failed to prevent cross talk in this experiment, we decided to investigate this further.

Several features of these two knock-in constructs favor the detection of ON-to-OFF cross talk. First, both gRNAs target the same position in the reading frame of their respective target genes (frame +1). Second, Cre_OFF_ GluA1-Halo targets the GluA1 C terminus with a stop codon in the Halo donor, while Cre_ON_ Flp_OFF_ smFP_V5_-Ca_V_2.3 targets the Ca_V_2.3 N terminus. Thus, ON-to-OFF cross talk would lead to an in-frame addition of smFP_V5_ in *Gria1*, while OFF-to-ON cross talk would introduce an early stop codon in *Cacna1e*, likely preventing protein expression from the allele.

To test whether cross talk could be prevented, we first titrated the amount of Cre_OFF_ GluA1-Halo vector. Unexpectedly, we found that higher Cre_OFF_ GluA1-Halo load increased the number of GluA1-Halo knock-in cells (although the effect was not obvious in statistical analysis because of high variability between cultures; Fig. 5*D*, *p *=* *0.21, one-way ANOVA), the opposite of what we previously found for Cre_OFF_ β3-tubulin (Fig. 3*A*). Higher Cre_OFF_ GluA1-Halo load also appeared to increase the number of double knock-in cells and incorrect knock-ins (Cre_ON_ Flp_OFF_ smFP_V5_-Ca_V_2.3 *p *=* *0.80, double knock-ins *p *=* *0.26, incorrect knock-ins *p = *0.63). At 38 fmol Cre_OFF_ vector, ∼25% of all fluorescent cells were incorrect (predominantly ON-to-OFF cross talk).

We hypothesized that this cross talk is because of a low editing rate of *Gria1* compared with genes with lower rates of cross talk (e.g., *Tubb3* and *Actb*; Fig. 3), which continues after Cre activation at DIV 7. Indeed, several studies demonstrated that both the appearance of DSBs, as well as (DNA-repair dependent) indels are highly dependent on the sequence of the target locus, and that repair of DSBs may continue for multiple days (Rose et al., 2017; Liu et al., 2020; Park et al., 2021). To compare editing rates over time, we transfected Cre_OFF_ GFP knock-ins for β3-tubulin, β-actin, and GluA1 at DIV 2, and fixed and counted the number of GFP-positive cells at DIV 4, 7, 10, and 14 (Fig. 5*E*). For all knock-ins, we found that the number of GFP-positive cells increased over time (*p *=* *1.0 × 10^−5^, two-way ANOVA), and this effect differed between knock-in constructs (*p *=* *5.0 × 10^−5^, interaction *p *=* *0.012, two-way ANOVA). Importantly, while the number of β3-tubulin and β-actin knock-in neurons increased at a similar rate (*p *=* *0.81, two-way ANOVA *post hoc* comparison), the number of GluA1 knock-in neurons increased much slower (β3-tubulin vs GluA1 *p *=* *0.0006, β-actin vs GluA1 *p *=* *3.5 × 10^−3^, two-way ANOVA *post hoc* comparison) and continued to increase after DIV 10.

Finally, we tested whether delaying Cre activation, by delaying the addition of 4OH-tamoxifen, would reduce ON-to-OFF cross talk (Fig. 5*G*). We found that delaying 4OH-tamoxifen addition had no effect on the number of single or double knock-in neurons (Cre_OFF_ GluA1-Halo *p *=* *0.91, Cre_ON_ Flp_OFF_ smFP_V5_-Ca_V_2.3 *p *=* *0.17, double knock-ins *p *=* *0.59, one-way ANOVA), but clearly diminished the number of incorrect knock-in cells (*p *=* *0.041, one-way ANOVA). Thus, while editing of *Gria1* is much slower compared with other genes, cross talk can be largely reduced by delayed activation of Cre.

### CAKE enables dual-color SMLM of endogenous synaptic proteins

Mapping the localization of endogenous synaptic proteins is crucial for our understanding of the brain (Choquet et al., 2021). In particular, deciphering which proteins regulate AMPA receptor nanoscale clustering in glutamatergic synapses will be pivotal in understanding synaptic transmission. Interestingly, AMPA receptors have been shown to be concentrated in subsynaptic PSD95 nanodomains (MacGillavry et al., 2013; Nair et al., 2013). Here, we used CAKE to uncover the nanoscale co-organization of endogenously tagged PSD95 and the AMPA receptor subunit GluA1 at glutamatergic synapses with dual-color SMLM.

**Figure 6.**
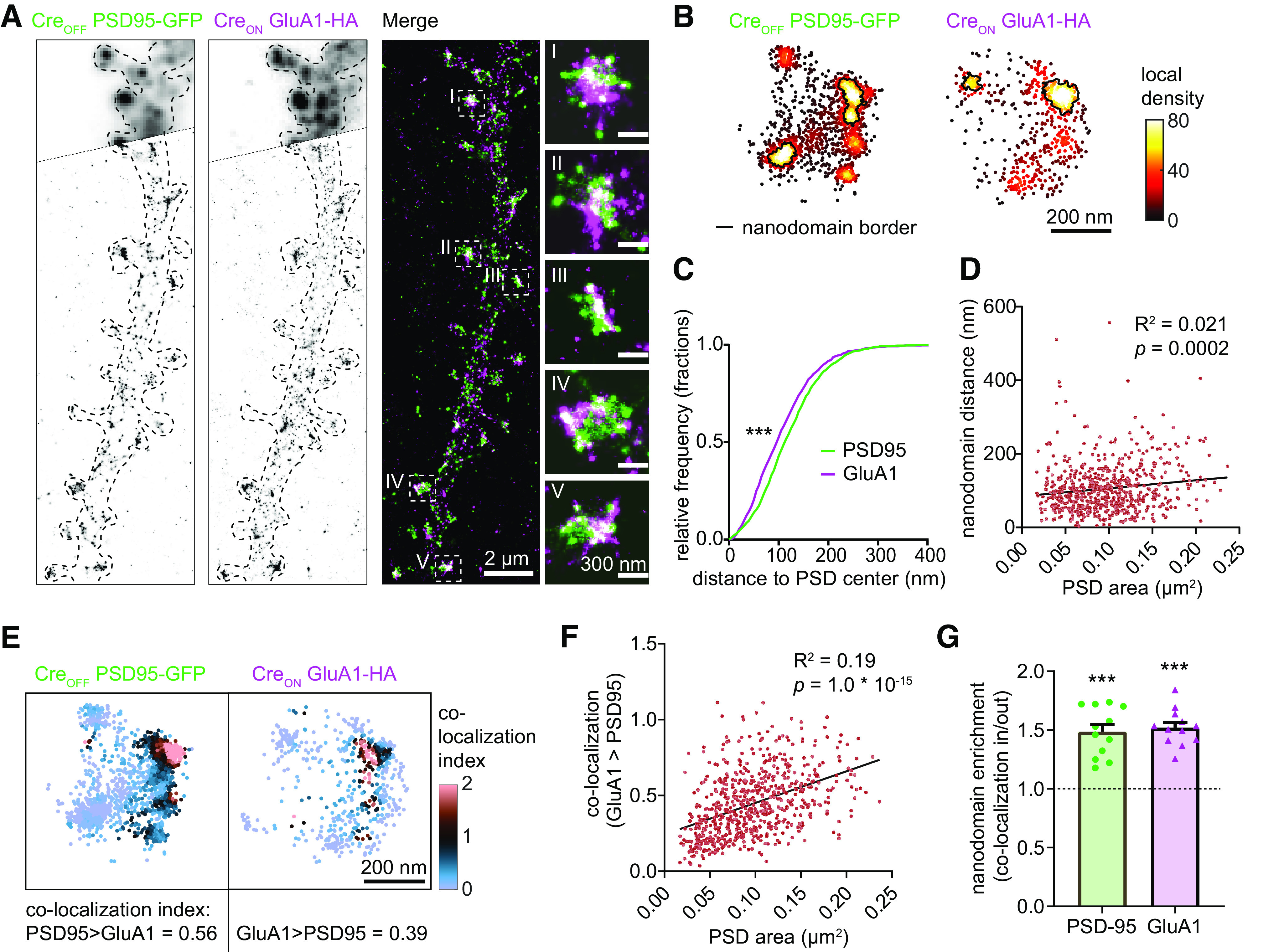
Dual-color SMLM reveals nanoscale co-organization of endogenous PSD95 and GluA1. ***A***, SMLM reconstruction of PSD95-GFP and GluA1-HA. Top part of the two channels shows widefield image of the same dendrite. Pixel size of reconstruction: 12 × 12 nm. ***B***, Example synapse showing the local density values of PSD95 and GluA1 localizations. Nanodomains are outlined with a black line. ***C***, Nanodomain topology of PSD95 and GluA1 nanodomains. Cumulative distributions of the distance between nanodomain and PSD center for both PSD95 (green) and GluA1 (magenta). GluA1 nanodomains reside on average 14 nm closer to the PSD center; ***p = 5.2 × 10^−8^, Mann–Whitney test. 1285 nanodomains were analyzed for PSD95 and 774 nanodomains were analyzed for GluA1. *n* = 12 neurons, *N* = 4 independent cultures. ***D***, Distance between PSD95 and GluA1 nanodomains correlates weakly with PSD size. PSD size is based on the cluster of PSD95 localizations inside the synapse (see Materials and Methods). *R*^2^ = 0.021, *p* = 0.0002, model linear regression (black line). A total of 656 synapses were analyzed. *n* = 12 neurons, *N* = 4 independent cultures. ***E***, Example synapse showing the co-localization index of PSD95 and GluA1 localizations. The average co-localization index of this synapse is indicated below the graph. See Extended Data Figure 6-1*D* for the co-localization indices of all imaged cells and synapses. ***F***, The co-localization of GluA1 with PSD95 localizations correlates with PSD size. *R*^2^ = 0.192, *p* = 1.0 × 10^−15^, model linear regression (black line). A total of 656 synapses were analyzed. *n* = 12 neurons, *N* = 5 independent cultures. ***G***, PSD95 and GluA1 are both enriched in nanodomains of the other channel. Relative co-localization of PSD95 and GluA1 inside versus outside nanodomains is plotted. Dotted line represents null hypothesis (no nanodomain enrichment). PSD95 ***p = 6.1 × 10^−6^, GluA1 ***p = 1.6 × 10^−7^, one sample *t* test.

As expected, endogenously tagged PSD95 and GluA1 co-localized at synapses (Fig. 6*A*). Using custom local density-based cluster analysis (based on Chen et al., 2020; MacGillavry et al., 2013), we identified nanodomains of both GluA1 and PSD95 within individual synapses (Fig. 6*B*). Synapses contained on average more PSD95 nanodomains, but larger GluA1 nanodomains (Extended Data Fig. 6-1*A*,*B*, nanodomain number *p *= 2.0 × 10^−4^, nanodomain diameter *p *=* *6.4 × 10^−8^, unpaired *t* test). These GluA1 nanodomains occurred on average only 14 nm closer to the center of the PSD (Fig. 6*C*, *p *=* *5.2 × 10^−8^, unpaired *t* test), and the distance between PSD95 and GluA1 nanodomains only slightly increased with larger PSDs (Fig. 6*D*, *p *=* *0.0002, *R*^2^ = 0.021, model linear regression). This suggests that PSD95 and GluA1 have similar synaptic topologies across synapse sizes. To further assess the co-organization of PSD95 and GluA1, we used a local density-based co-localization index (Fig. 6*E*; Extended Data Fig. 6-1*C*; see Materials and Methods for details). Individual synapses had highly variable degrees of co-localization of PSD95 and GluA1 (Extended Data Fig. 6-1*C*). In addition, the co-localization of PSD95 and GluA1 was correlated with PSD size (Fig. 6*F*, *p *=* *1.0 × 10^−15^, *R*^2^ = 0.19), suggesting that stronger synapses have a tighter association between GluA1 and PSD95. Lastly, we observed that co-localization between GluA1 and PSD95 was higher inside nanodomains compared with the rest of the PSD (Fig. 6*G*, PSD95 *p *=* *6.1 × 10^−6^, GluA1 *p *=* *1.6 × 10^−7^, one-sample *t* test), confirming previous observations (MacGillavry et al., 2013; Nair et al., 2013). In summary, CAKE allowed us to reveal the tight nanoscale co-organization of endogenous PSD95 and AMPA receptors, which correlated with synapse size.

10.1523/ENEURO.0056-22.2022.f6-1 Extended Data Figure 6-1 PSD95 and GluA1 have distinct nanoscale organizations. ***A***, Number of nanodomains of PSD95 and GluA1 per PSD; ***p = 0.0002, unpaired *t* test. ***B***, Nanodomain diameter for PSD95 and GluA1; ***p = 6.4 × 10^−8^, unpaired *t* test. ***C***, Nanodomain diameter for PSD95 and GluA1 per PSD. Dotted line represents minimal threshold of nanodomain diameter. ***D***, Co-localization index for both PSD95 and GluA1, plotted per synapse (transparent red dots) and neuron (red dots). Download Figure 6-1, EPS file.

### Acute immobilization of endogenous synaptic AMPA receptors using inducible hetero-dimerization

**Figure 7.**
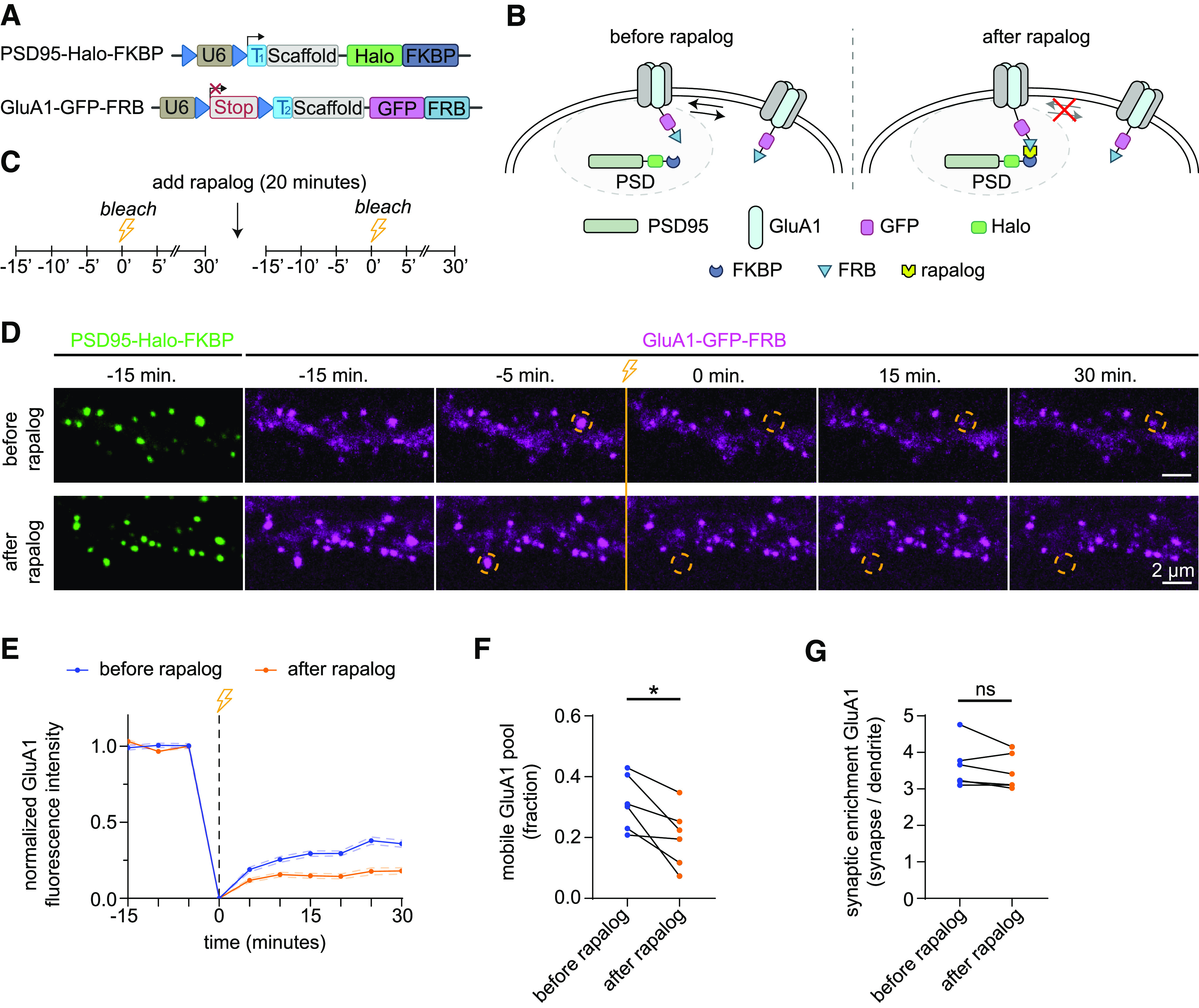
Live-cell modulation of endogenous AMPA receptor anchoring using CAKE. ***A***, Overview of DNA constructs used. ***B***, Graphical overview of synaptic anchoring of AMPA receptors using rapalog. FRB (fused to GluA1), binds to FKBP (fused to PSD95), preventing exchange of synaptic receptors. ***C***, Imaging protocol. Neurons are imaged every 5 min using spinning disk confocal microscopy. FRAP is performed twice, before and after the incubation with rapalog for 20 min. ***D***, Example FRAP acquisition before and after incubation with rapalog. GluA1-GFP-FRB in magenta and PSD95-Halo-FKBP in green. Spines indicated with orange circle are bleached just before time point 0 min. ***E***, FRAP curves of spines bleached before and after incubation with rapalog. Data are normalized to the average intensity before bleaching. A total of 63 spines before rapalog and 66 spines after rapalog were bleached. *n* = 6 cells, *N* = 5 independent cultures. ***F***, Average recovery of fluorescence per neuron, averaged over the last four frames, reflecting the mobile pool of receptors. After rapalog, the mobile pool of receptors is less than before rapalog; *p = 0.018, paired *t* test. *n* = 6 cells, *N* = 5 independent cultures. This experiment was repeated for Cre_ON_ GluA1-GFP-FRB single knock-ins (Extended Data Fig. 7-1). ***G***, Synaptic enrichment of GluA1-GFP-FRB at synapses before and after rapalog incubation are similar. Synaptic enrichment is the relative fluorescence intensity at the synapse compared with the dendrite. *p* = 0.21, paired *t* test. *n* = 6 cells, *N* = 5 independent cultures.

Labeling of two endogenous proteins simultaneously in single neurons is a powerful means to measure and modulate their (functional) co-localization, but experiments are often hampered by overexpression artifacts. For example, the amount of PSD95 as well as AMPA receptors determine synaptic strength, and overexpression of these synaptic components can result in altered synaptic function (El-Husseini et al., 2000; Schnell et al., 2002). Interestingly, precise control of lateral diffusion and synaptic exchange of AMPA receptors is important for both basal transmission and synaptic plasticity (Groc and Choquet, 2020). Thus, studying the trafficking and anchoring of AMPA receptors requires methods that accurately modulate the localization of endogenous proteins in living neurons. Here, we used CAKE to label AMPA receptors and PSD95 with inducible dimerization modules to acutely manipulate AMPA receptor anchoring at the synapse. More specifically, we generated C-terminal CAKE knock-in constructs to label GluA1 and PSD95 with the rapalog-inducible dimerization domains FRB and FKBP, respectively (Fig. 7*A*; Kapitein et al., 2010a). Addition of rapalog would then anchor AMPA receptors to PSD95 proteins at the synapse, thus reducing receptor exchange between the PSD and the extrasynaptic membrane (Fig. 7*B*).

10.1523/ENEURO.0056-22.2022.f7-1 Extended Data Figure 7-1 Live-cell anchoring of AMPA receptors control. ***A***, Overview of DNA construct used. ***B***, Imaging protocol. Neurons are live-cell imaged using spinning disk microscopy every 5 min. FRAP is performed twice, before and after vehicle incubation for 20 min. ***C***, FRAP curves of spines bleached before and after vehicle incubation. Data are normalized to the average intensity before bleaching. A total of 34 spines before vehicle and 42 spines after vehicle were bleached. *n* = 6 cells, *N* = 5 independent cultures. ***D***, Average recovery of fluorescence per neuron, averaged over the last four frames, reflecting the mobile pool of receptors. No difference was observed before and after vehicle. *p* = 0.86, paired *t* test. *N* = 6 cells, *N* = 5 independent cultures. Download Figure 7-1, EPS file.

We performed live-cell spinning disk imaging experiments to measure the FRAP at synapses to quantify GluA1 turnover under basal conditions and after the addition of rapalog (Fig. 7*C*,*D*). Under basal conditions, the fluorescence recovery of GluA1 receptors was 0.31 ± 0.04 (Fig. 7*E*,*F*), which is consistent with previous studies (Chen et al., 2021; Fang et al., 2021). We incubated neurons with rapalog for 20 min, and measured FRAP dynamics on a different dendrite of the same neuron. Rapalog induced a strong decrease in fluorescence recovery (0.20 ± 0.04) of GluA1, indicating successful anchoring of AMPA receptors to PSD95 (Fig. 7*E*,*F*, *p *=* *0.018, paired *t* test). Importantly, we did not observe a decrease of GluA1 turnover in neurons that were not treated with rapalog (Extended Data Fig. 7-1, *p *=* *0.86, paired *t* test). We found no significant change in the enrichment of GluA1 at the synapse after rapalog (Fig. 7*G*, *p *=* *0.21, paired *t* test). Together, these results show that CAKE allows for labeling and rapidly inducible dimerization of endogenous proteins in living neurons.

## Discussion

Accurate detection and manipulation of endogenous proteins is essential to understand cell biological processes, which motivated laboratories across cell biology to develop highly efficient CRISPR genome editing methods for endogenous epitope tagging (Auer et al., 2014; Nakade et al., 2014; Lackner et al., 2015; Schmid-Burgk et al., 2016; Suzuki et al., 2016; Nishiyama et al., 2017; Artegiani et al., 2020; Danner et al., 2021). Multiplex editing using NHEJ-based CRISPR/Cas9 methods remains limited because of the high degree of cross talk that occurs between two knock-in loci (Gao et al., 2019; Willems et al., 2020). In the current study we present CAKE, a mechanism to diminish cross talk between NHEJ-based CRISPR/Cas9 knock-ins using sequential activation of gRNA expression. We demonstrate that this mechanism strongly reduces cross talk between knock-in loci, and results in dual knock-ins for a wide variety of genes. Finally, we showed that CAKE can be directly applied to reveal new biological insights. CAKE allowed us to perform two-color super-resolution microscopy and acute manipulation of the dynamics of endogenous proteins in neurons, together revealing new insights in the nanoscale organization of synaptic proteins.

### Factors that influence knock-in efficacy

The CAKE mechanism presented here creates a mosaic of Cre_ON_ and Cre_OFF_ knock-ins, and the number of double knock-in cells depends on the efficacy of each knock-in vector. Therefore, to obtain a high number of double knock-in cells, the efficacy of both the Cre_ON_ and Cre_OFF_ knock-in vector must be optimized. We identified three parameters that regulate the efficacy for single and double knock-ins in neurons. First, the efficacy of gRNAs varies widely, and even gRNAs that target sequences a few base pairs apart in the same locus can have dramatically different knock-in rates (Willems et al., 2020; Danner et al., 2021; Fang et al., 2021; Zhong et al., 2021). Thus, the efficacy of each individual gRNA must be optimized to increase the chance of successful multiplex labeling in neurons. gRNA performance is dependent on many factors, including the rate of DNA cleavage and repair (Rose et al., 2017; Liu et al., 2020; Park et al., 2021) and the propensity of the target locus for indel mutations (Rose et al., 2017; Shen et al., 2018; Liu et al., 2020). While some of these parameters can be predicted computationally (Doench et al., 2014; Shen et al., 2018; Park et al., 2021), the efficacy of each individual gRNA should be verified experimentally. We performed most experiments with gRNAs that we and others previously found to yield a high number of single knock-ins (Suzuki et al., 2016; Willems et al., 2020).

Second, we found knock-in efficacy to be highly sensitive to knock-in vector amount. For multiple knock-ins, we found that reducing the amount of knock-in vector increased the number of knock-in-positive cells, with the optimum for β3-tubulin as little as 3.9 fmol vector DNA per coverslip. We propose that this inverse relationship is because of competition between donor DNA molecules for integration. In line with these observations, knock-in vectors with multiple donors reduced the number of β3-tubulin-GFP-positive cells. Donor competition could also explain why we consistently needed more knock-in vector for the Cre_ON_ knock-in, as remaining donor from the Cre_OFF_ vector could compete for integration in the target locus after Cre activation. Importantly, for reasons incompletely understood, the optimum vector amount differs considerably between knock-in constructs. A striking example in this respect is GluA1, which requires a 10- to 50-fold high vector load to reach the maximum number of knock-in cells. We also observed that onset of GluA1 knock-in expression is much slower compared with other genes, and thus there may be a relationship between the editing rate of the targeted locus, and the amount of donor DNA required for successful integration.

Third, the timing of Cre expression and activation may influence the number of incorrect knock-in cells. For Cre_OFF_ GluA1/Cre_ON_ Ca_V_2.3 we found that delaying the activation of ER^T2^-Cre-ER^T2^ by 7 d diminished cross talk between the knock-ins. This is in line with our observation that GluA1 knock-ins are completed at a much slower rate than other genes tested here. We did not observe an effect of infection day for lenti-Cre with Cre_OFF_ β3-tubulin and Cre_ON_ β-actin. However, the slow onset of expression from lentiviral vectors (in the order of days; Hioki et al., 2007) makes lentivirus a weak method to observe an effect Cre timing, in particular compared with the rapid activation of ER^T2^-Cre-ER^T2^ (Matsuda and Cepko, 2007). Additionally, donor integration in *Tubb3* appears to be relatively fast, which limits the window for cross talk to occur.

### Comparison of CAKE with other CRISPR/Cas9 strategies

Previous studies for NHEJ-based multiplex knock-ins had restrictions on donor DNA design and the target loci that could be combined (e.g., N-terminal or C-terminal knock-ins), and these methods could inadvertently reduce protein expression from the targeted allele (Gao et al., 2019; Willems et al., 2020). The CAKE knock-in strategy presented here lifts these limitations, and can be used with any locus and donor DNA design. Importantly, because CAKE only relies on sequential gRNA expression, it is expected to be compatible with any combination of NHEJ-based knock-in modalities.

Multiple CRISPR/Cas9 modalities have been developed to generate knock-ins in neuronal preparations, predominantly based on NHEJ. The first generation of tools, including HITI (Suzuki et al., 2016), ORANGE (Willems et al., 2020), and HiUGE (Gao et al., 2019) directly edit the coding sequence of genes to insert a fluorophore or epitope tag. This simplifies design and limits the number of cloning steps required, at the expense of potential indel mutations that disrupt the reading frame (Suzuki et al., 2016; Willems et al., 2020). HiUGE further simplifies design by using a universal donor DNA, but requires the expression of an extra gRNA to make the donor available for integration (Gao et al., 2019). All three methods leave a small scar of a few base pairs after knock-in completion. MMEJ-based techniques yield seamless knock-ins by using homology between the donor and target gene (Yao et al., 2017). This homology, however, is unlikely to have a major effect in preventing double knock-in cross talk: because an MMEJ-donor is blunt ended, it can integrate in any DSB. Second generation NHEJ-based techniques are scarless and are relatively insensitive to indels, by replacing exons (TKIT; Fang et al., 2021) or introducing novel exons (CRISPIE; Zhong et al., 2021). While the cloning of CRISPIE knock-ins is more straightforward compared with TKIT, it may be challenging to find a suitable location for integrating a novel exon, without disrupting protein folding or function. Finally, the recently proposed PASTE strategy obtains single and multiplex knock-ins without creating DSBs, and might be applicable in neurons as well (Ioannidi et al., 2021).

### Application of CAKE for labeling and manipulation of endogenous proteins

CAKE opens possibilities to study localization, mobility and function of endogenous proteins at multiple levels. For instance, the nanoscale organization of synaptic proteins profoundly influences information transfer at synapses (MacGillavry et al., 2013; Nair et al., 2013; Tang et al., 2016; Rebola et al., 2019), and CAKE may present an invaluable tool to decipher synapse organization using endogenous protein labeling under stringent conditions required for super-resolution microscopy. Indeed, we were able to accurately determine the subsynaptic co-localization of GluA1 and PSD95, which scaled linearly with PSD size. This suggests that AMPAR confinement at PSD95 clusters is associated with synapse strength, since PSD size is correlated with synaptic weight (Holler et al., 2021). To study protein-protein interactions CAKE could also be applied in combination with Förster resonance energy transfer reporters, bimolecular fluorescence complementation (Tebo and Gautier, 2019), or proximity biotinylation assays (De Munter et al., 2017).

Our results also illustrate the potential of CAKE to manipulate the localization, mobility and functionality of endogenous proteins, for instance by recruiting or anchoring proteins and organelles in living cells using optogenetic or chemical dimerization modules. Trafficking and subcellular positioning of proteins is crucial for all cellular processes, and this unique combination of techniques allows manipulation of endogenous protein dynamics in cells. For example, directed positioning of receptors (Sinnen et al., 2017) or even entire organelles (Van Bergeijk et al., 2015) have previously been shown to influence synaptic strength and neuronal development, respectively.

The method of Cre expression or activation can be tailored to experimental needs, and besides viral Cre delivery or ER^T2^-Cre-ER^T2^, users can consider for instance doxycycline-dependent Cre, TAT-Cre or a mouse line that expresses Cre. Regardless of the method, the timing of Cre activity should be carefully characterized, to prevent cross talk between Cre_OFF_ and Cre_ON_ knock-ins. The conditional activation of CRISPR/Cas9 knock-ins opens new avenues for detailed analysis of endogenous proteins in individual cell types. By restricting Cre or Flp expression using cell-type specific promoters (Taniguchi et al., 2011; He et al., 2016), combined with Cre_ON_ or Flp_ON_, one could map out the spatiotemporal expression of proteins in a wide variety of cells at unprecedented precision.

Taken together, we created and validated a series of CRISPR tools for sequential genome editing to create multiplex knock-ins with NHEJ, and demonstrate the value of these tools for determining and manipulating protein distribution in neurons.

## Synthesis

Reviewing Editor: Deanna Smith, University of South Carolina Decisions are customarily a result of the Reviewing Editor and the peer reviewers coming together and discussing their recommendations until a consensus is reached. When revisions are invited, a fact-based synthesis statement explaining their decision and outlining what is needed to prepare a revision will be listed below. The following reviewer(s) agreed to reveal their identity: Erin Schuman. Note: If this manuscript was transferred from JNeurosci and a decision was made to accept the manuscript without peer review, a brief statement to this effect will instead be what is listed below.

Both reviewers indicated that this work is of high quality and that CAKE will be an important advance in the field of synaptic biology. The manuscript describes methods that allow investigators to tag more than one protein in individual neurons and guides the user on how to optimize their experimental conditions. Endogenous protein tagging is an improvement over overexpression of tagged synaptic proteins which is usually phenotypic, and thus provides an advance in the field. Reviewers made a few comments which should be addressed in the revision.

Reviewer 1:

In the manuscript titled ’Multiplex labeling and manipulation of neuronal proteins using sequential CRISPR/Cas9 gene editing’ Droogers and colleagues present a technique for sequential endogenous tagging at different genomic loci. The advantage of the proposed methodology, termed ’Conditional Activation of Knock-in Expression (CAKE)’, is that, despite still relying on NHEJ to achieve knock-in, the temporal control capabilities built into the system reduce the rates of spurious tagging. The authors present a remarkable body of validation and optimization so that a potential user could expect to implement this approach in their lab with minimal effort. The helpful schematic in Figure S3 thoroughly illustrates the CAKE vector design process, making the adaptation of this approach to any target of interest relatively straightforward. Lastly, the authors demonstrate how this approach can be used to study the relative distribution of synaptic proteins, and to engineer an oligomerization system to perturb AMPA receptor anchoring. The work presented is very extensive, of high quality and certainly suitable for publication in eNeuro. As a service to the community and also to ensure that the system is widely accessible and used the authors should make all constructs available through Addgene. In addition, the authors should address the comments outlined below:

• The title should be changed to ‘duplex labelling’ as only duplex labelling was demonstrated and it’s difficult to imagine how a higher degree of multiplexing could be achieved with the current methodology.

• Line 72: The authors state: ’... and will generate null mutations for N-terminally labeled proteins when the incorrect donor is integrated’. Do the authors intend to say ’... will generate null mutations for N terminally labelled proteins when the donor template is integrated incorrectly’? ORF disruption could occur in a scenario where the correct template integrates incorrectly but also when the incorrect template integrates at the site, this should be explained more clearly.

• The schematic for Cre delivery outlined in Figure 1C should include the alternative methods for Cre delivery.

• Have the authors tried using TAT-Cre in their experiments?

• Line 103: The authors state: ‘Second, to reduce crosstalk, we redesigned the first knock-in vector such that gRNA expression of the first vector is switched off by Cre, effectively limiting further editing of the first targeted locus’. Would this make any difference considering that knock-in is expected to disrupt the PAM site after editing of the first locus?

• Line 178: The authors state: ‘... suggesting that either knock-in efficacy for this gene combination is insensitive to Cre timing, or that the onset of lentiviral mediated Cre expression is too slow to observe an effect’. Shouldn’t it be ‘the onset of lentiviral mediated Cre expression is too fast to observe an effect’?

• Line 212: ‘... which ensures rapid onset of Cre activation (Matsuda & Cepko, 2007) and superior control over timing’ should be changes to ‘over time’.

• In the section of the discussion where the CAKE system is compared to other CRISPR-Cas9 endogenous tagging strategies the authors should discuss MMEJ methods to achieve tagging. In this regard, would the short homology arms of an MMEJ template be sufficient to overcome spurious tagging or this would still occur by NHEJ? Can the authors comment on this point specifically?

Reviewer 2:

This manuscript by Droogers et al. presents CAKE, an optimized CRISPR/Cas9 gene editing strategy enabling endogenous tagging of two different genes (double knock-in) in individual neurons. The strategy is based on sequential knock-in using Cre or Flp recombinase and inducible vectors. It has been optimized to reduce the occurrence of errors in editing (“knock-in crosstalk”). The authors identify different parameters that affect double knock-in efficacy and optimize them for selected pairs of genes. They then demonstrate that their approach can be used for live-cell imaging and super-resolution microscopy of endogenous synaptic proteins (GluA1 and PSD95), as well as for the modulation of receptor-scaffold interactions.

Imaging endogenous proteins and various molecular species at the same time is a major challenge in neuroscience and this paper provides an interesting solution. My opinion is that this manuscript suitable for publication in eNeuro, provided small modifications of the text as suggested below:

(1) Overall the efficacy of double knock-in cells is (very) low and it would be useful to know the percentage of double knock-in cells among all transfected or single knock-in cells for each knock-in pair they have tested.

(2) In terms of methodology, the authors should explain step by step how they design, choose and validate gRNA sequences for the new genes knocked-in in this study. This will be the most difficult part for people who want to use CAKE for their genes of interest and providing step by step guidelines on how and where to knock-in a gene will be a major plus.

(3) The authors mention previous knock-in approaches developed by others. They should explain their basis and limitation so that the manuscript is more accessible to scientists that are not experts in Crispr knock-in techniques.

## Author Response

Response to reviewer comments

First and foremost, we would like convey our gratitude to both reviewers for their thorough review of the manuscript and the constructive suggestions. We have made multiple improvements to the manuscript based on the reviewers feedback.

Firstly, the title has been changed , (“Duplex labelling” ... instead of “Multiplex labelling...”) according to the suggestion of Reviewer 1. Secondly, to make CAKE more accessible to readers unfamiliar with CRISPR/Cas9-mediated gene editing we added a section to the main text to discuss CAKE in the context of other CRISPR/Cas9 modalities. This includes suggestions on how multiple techniques can be combined to generate duplex knock-ins. Thirdly, we included step-by-step guidelines to design and clone CAKE knock-in vectors. Finally, we made multiple modifications to the main text to improve clarity and to provide technical details, such as the double knock-in efficiency in our optimization experiments. All changes are highlighted as green text and the parts that have been replaced are highlighted as red, strikethrough text.

In conclusion, we feel that these modifications improve the manuscript significantly, and increase its scope for a broader audience. Below we have provided point-by-point replies to the comments of the reviewers.

Reviewer 1:

In the manuscript titled ’Multiplex labeling and manipulation of neuronal proteins using sequential CRISPR/Cas9 gene editing’ Droogers and colleagues present a technique for sequential endogenous tagging at different genomic loci. The advantage of the proposed methodology, termed ’Conditional Activation of Knock-in Expression (CAKE)’, is that, despite still relying on NHEJ to achieve knock-in, the temporal control capabilities built into the system reduce the rates of spurious tagging. The authors present a remarkable body of validation and optimization so that a potential user could expect to implement this approach in their lab with minimal effort. The helpful schematic in Figure S3 thoroughly illustrates the CAKE vector design process, making the adaptation of this approach to any target of interest relatively straightforward. Lastly, the authors demonstrate how this approach can be used to study the relative distribution of synaptic proteins, and to engineer an oligomerization system to perturb AMPA receptor anchoring. The work presented is very extensive, of high quality and certainly suitable for publication in eNeuro. As a service to the community and also to ensure that the system is widely accessible and used the authors should make all constructs available through Addgene. In addition, the authors should address the comments outlined below:

We would like to thank the reviewer for the in-depth review of the manuscript and the constructive comments and suggestions that were provided. All constructs will be made available through Addgene as requested, and Addgene IDs are added to Table 2.

• The title should be changed to ‘duplex labelling’ as only duplex labelling was demonstrated and it’s difficult to imagine how a higher degree of multiplexing could be achieved with the current methodology.

We agree with the reviewer that ‘Duplex labelling’ is more correct, since the efficiency that we currently achieve is not high enough for generating a higher degree of multiplexing. The title has been adapted accordingly.

• Line 72: The authors state: ‘... and will generate null mutations for N-terminally labeled proteins when the incorrect donor is integrated’. Do the authors intend to say ‘... will generate null mutations for N terminally labelled proteins when the donor template is integrated incorrectly’? ORF disruption could occur in a scenario where the correct template integrates incorrectly but also when the incorrect template integrates at the site, this should be explained more clearly.

We agree with the reviewer that the text can lead to some confusion. We modified the text as follows: ‘... and will generate null mutations for N-terminally labeled proteins when the incorrect donor is integrated’ to ‘... and indel mutations or integration of the incorrect donor may generate null mutations’ (Lines 107-109).

• The schematic for Cre delivery outlined in Figure 1C should include the alternative methods for Cre delivery.

While we sympathize with the idea to include all experimental elements in one figure, we feel that adding Cre delivery to Figure 1C will distract the reader from the main message. Figure 1 is intended to explain the mechanism of CAKE, which is the major innovation of this manuscript. Adding methods to deliver Cre would add distraction from this, also because Cre delivery is discussed in detail only in Figure 4. Finally, it may suggest to some readers that CAKE does not work with other Cre delivery methods, (e.g. transgenic mouse lines, TAT-Cre, or doxicyclin-dependent Cre, AAV-Cre). We added a reference to Figure 4 in the figure caption. Furthermore, we added a section to the main text to discuss alternative Cre delivery systems (line 749-753).

• Have the authors tried using TAT-Cre in their experiments?

Our experiments are based on lenti-Cre or ERT2-Cre-ERT2, but we agree that there are many more options available to time the delivery of active Cre to your preparation. Therefore, we added a description of alternative methods for Cre delivery in the discussion (lines 749-753).

• Line 103: The authors state: ‘Second, to reduce crosstalk, we redesigned the first knock-in vector such that gRNA expression of the first vector is switched off by Cre, effectively limiting further editing of the first targeted locus’. Would this make any difference considering that knock-in is expected to disrupt the PAM site after editing of the first locus?

We agree with the reviewer that in an ideal situation the first locus cannot be edited after correct integration of the donor. However, recent studies suggest that editing of some loci can be rather slow, (up to multiple days, see main text line 556-559), and our data suggests this as well (Figure 5). Furthermore, the repair of DSBs is highly context-dependent, and some DSBs are repaired without indels (see for instance Shen et al, 2018 PMID 30405244 and Liu et al, 2020, PMID 32527834), thus allowing multiple rounds of DSB generation and repair. Thus, it is conceivable that, upon Cre delivery, the first locus can still be edited if the first target sequence has not been cleaved yet or that donor integration was unsuccessful. Therefore, to minimize incorrect knock-ins it is safest to disable expression of the first gRNA.

• Line 178: The authors state: ‘... suggesting that either knock-in efficacy for this gene combination is insensitive to Cre timing, or that the onset of lentiviral mediated Cre expression is too slow to observe an effect’. Shouldn’t it be ‘the onset of lentiviral mediated Cre expression is too fast to observe an effect’?

As described in Hioki et al. (2007), the onset of lentiviral Cre expression is in the order of days. Thus, even with lentiviral infection at DIV3, Cre expression might only peak at DIV5-7, covering the complete window of this experiment. It also shows that with lentivirus, we have relatively little control over the exact timing of Cre expression, and recombination of LoxP sequences, in each individual cell. We therefore concluded that this Cre delivery method is probably too slow to observe an effect of the timing of Cre infection.

• Line 212: ‘... which ensures rapid onset of Cre activation (Matsuda & Cepko, 2007) and superior control over timing’ should be changes to ‘over time’.

We agree with the reviewer that we were not clear enough in this instance. We therefore changed ’... and superior control over timing’ to ‘... and superior control over the timing of Cre activity’ (line 509).

• In the section of the discussion where the CAKE system is compared to other CRISPR-Cas9 endogenous tagging strategies the authors should discuss MMEJ methods to achieve tagging. In this regard, would the short homology arms of an MMEJ template be sufficient to overcome spurious tagging or this would still occur by NHEJ? Can the authors comment on this point specifically?

MMEJ yield seamless knock-ins at the targeted locus, due to microhomology between locus and donor sequence. However, it will most likely not overcome spurious tagging in untargeted loci, because an MMEJ donor in itself is blunt ended and can therefore integrate in any DSB via NHEJ. We added this piece of information to the evaluation of other CRISPR/Cas9 modalities in the discussion (lines 718-721).

Reviewer 2:

This manuscript by Droogers et al. Presents CAKE, an optimized CRISPR/Cas9 gene editing strategy enabling endogenous tagging of two different genes (double knock-in) in individual neurons. The strategy is based on sequential knock-in using Cre or Flp recombinase and inducible vectors. It has been optimized to reduce the occurrence of errors in editing (“knock-in crosstalk”). The authors identify different parameters that affect double knock-in efficacy and optimize them for selected pairs of genes. They then demonstrate that their approach can be used for live-cell imaging and super-resolution microscopy of endogenous synaptic proteins (GluA1 and PSD95), as well as for the modulation of receptor-scaffold interactions.

We want to thank the reviewer for in-depth review and the helpful suggestions for our manuscript, specifically regarding the accessibility of the technique.

Imaging endogenous proteins and various molecular species at the same time is a major challenge in neuroscience and this paper provides an interesting solution. My opinion is that this manuscript suitable for publication in eNeuro, provided small modifications of the text as suggested below:

(1) Overall the efficacy of double knock-in cells is (very) low and it would be useful to know the percentage of double knock-in cells among all transfected or single knock-in cells for each knock-in pair they have tested.

We agree with the reviewer that the efficiency of double knock-ins is valuable information for researchers that consider adopting CAKE. We calculated and described the efficiency of double knock-ins for the knock-in pairs β3-tubulin and β-actin, and GluA1 and PSD95. The efficiencies were reported for the data shown in Fig. 3A, Fig. 3-1A, Fig. 4B and Fig. 4-1B. It is important to point out at the knock-in efficiency may vary considerably between preparations and is dependent on methodology used for DNA delivery and Cre activation (See for instance Figure 4). Therefore, one should always optimize and tailor experimental conditions to their own demands.

(2) In terms of methodology, the authors should explain step by step how they design, choose and validate gRNA sequences for the new genes knocked-in in this study. This will be the most difficult part for people who want to use CAKE for their genes of interest and providing step by step guidelines on how and where to knock-in a gene will be a major plus.

We agree with the reviewer that a detailed description of knock-in design will aid researchers in using CAKE for their own research. Therefore, we provide step-by-step guidelines for the generation of CAKE-compatible knock-in constructs. This protocol can be found as Extended Data 1-4. Furthermore, we added a new Table 3 with the design rationale of all knock-ins introduced in this study. Details on previously designed knock-ins, as well as a detailed discussion on the design of novel single knock-ins can also be found in Willems et al, 2020.

(3) The authors mention previous knock-in approaches developed by others. They should explain their basis and limitation so that the manuscript is more accessible to scientists that are not experts in Crispr knock-in techniques.

We agree with the reviewer that a description of other CRISPR/Cas9 modalities can make the CAKE approach more accessible. Therefore, we have extended the part in the discussion where we compare CAKE with other CRISPR/Cas9 modalities (lines 702-727).
