# Supplementary material for: Duplex Labeling and Manipulation of Neuronal Proteins Using Sequential CRISPR/Cas9 Gene Editing
Source: eNeuro. 2022 Jul 26;9(4):ENEURO.0056-22.2022. doi: 10.1523/ENEURO.0056-22.2022 (PMC9333357; doi:10.1523/ENEURO.0056-22.2022)
Supplement: Extended Data Figure 1-4 — Step-by-step guidelines for making ORANGE CAKE knock-ins, including genomic target sequence selection (A), design of primers for gRNA and donor DNA (A), and cloning into any CAKE template vector (B, C). Download Figure 1-4, DOCX file. [file enu-eN-MNT-0056-22-s02.docx]

# Step-by-step guidelines for design and cloning of ORANGE CAKE knock-in constructs

These step-by-step guidelines can be used to generate ORANGE CAKE (pOCx) knock-in vectors, and includes genomic target sequence selection, design of primers for guide RNA (gRNA) and donor DNA, and cloning into pOCx vectors. Cloning into pOC2 (Cre­_off_) is taken as example, but cloning steps are identical for all pOCx vectors. The cloning workflow is displayed in Figure 1-3B, and further examples of knock-in design are displayed in Figure S1 of Willems et al., 2020.

### (A) Target sequence selection and knock-in design

1. Perform a literature survey on the protein of interest, and identify a location in the protein that will most likely tolerate the addition of an epitope tag. Consult studies that previously developed recombinant fusion proteins with fluorescent proteins, or affinity-purification tags such as His or GST. Avoid labeling directly into protein domains or sequences with (predicted) α-helices or β-strands, and consider the presence of a signal peptide and critical post-translational modifications. UniProt can be used to identify these sequences. Most commonly, proteins are labeled at the N- or C-terminus, but some proteins tolerate labeling in (large) unstructured sequences or loops. If no literature is available, a C-terminal tag is usually preferred, as frame-shift mutations are less likely to be detrimental. Also see the methods section of Willems et al., 2020.
2. Retrieve the genomic sequence of the target gene from the [UCSC genome browser](http://genome-euro.ucsc.edu/index.html) and load it into a DNA handling tool such as SnapGene.
3. Select a few target sequences using the UCSC genome browser, CRISPick or CRISPOR (an overview of tools can be found [here](https://zlab.bio/guide-design-resources)). Target sequences should be as close as possible to the ideal integration site (for C-terminal tags just before the stop codon). Also consider specificity using the MIT Guide Specificity Score. Higher scores perform better, scores above 75 are therefore preferred. The UCSC genome browser also generates a list with likely off-target loci; avoid off-target loci in exons where possible.
4. Annotate the selected target sequences in SnapGene and determine the Cas9 cleavage site for each target sequence (3 bp upstream from the PAM sequence). This information is needed to determine the frameshift compensation in to donor (see step 5).
5. Design the donor for each target sequence according to the examples of Figure S1 of Willems et al., 2020 and the cloning guide in Figure 1-3B. Make sure that the following elements are included in the indicated order:
   1. HindIII restriction site (alternatively XbaI)
   2. reverse complement of genomic target sequence (including PAM)
   3. frameshift compensation (see table below)
   4. linker (e.g. Alanine-Serine-Glycine with BmtI restriction site)
   5. donor tag (e.g. HaloTag)
   6. linker (e.g. Serine-Alanine-Glycine with AfeI restriction site)
   7. frameshift compensation (see table below)
   8. reverse complement of genomic target sequence (including PAM)
   9. MluI restriction site (alternatively MunI)

*Guide for frameshift compensation:*

| Genomic PAM site on lagging strand  (5' **CCN** 3' in coding strand) | | | Genomic PAM site on coding strand  (5' **NGG** 3' in coding strand) | | |
| --- | --- | --- | --- | --- | --- |
|  | *number of bp to add…* | |  | *number of bp to add…* | |
| **Cas9 cuts at:** | **forward primer** | **reverse primer** | **Cas9 cuts at:** | **forward primer** | **reverse primer** |
| in frame | 0 | 1 | *in frame* | 1 | 0 |
| frame + 1 | 2 | 2 | *frame + 1* | 0 | 1 |
| frame + 2 | 1 | 0 | *frame + 2* | 2 | 2 |

1. Check for each target sequence in the designed donor if unwanted amino acids or stop codons are introduced after donor DNA integration. Unfavorable amino acids have side chains that are e.g. hydrophobic (Leucine, Methionine, Phenylalanine, Tryptophan and Tyrosine), electrically charged (Arginine and Histidine) or important for secondary and tertiary protein structure (Proline and Cysteine). If multiple undesired amino acids are present, consider using a different Cas9 target sequence.
2. Choose the best target sequence based on the criteria indicated above and design primers for the PCR of the full donor (See Willems et al., 2020 Figure S1). Make sure the primers have a minimal annealing temperature of 45°C.
3. Simulate knock-in: copy the donor sequence between the two Cas9 cleavage sites, and paste it in the genomic cleavage site. Double check that the donor is in frame at both 5’ and 3’ end, and that no stop codons are introduced.
4. Design primer pair for gRNA target sequence (example in Figure S1 of Willems et al., 2020). Place the target sequence on the coding strand (do not include PAM sequence). An extra G can be placed at 5’ of the target sequence for improved transcription initiation. Both forward and reverse primers have an overhang at 5’ to ensure insertion into BbsI restriction site. For pOC2, overhang of forward primer is 5’ ttgt 3’ and overhang of reverse primer is 3’ caaa 5’. Notice that these overhangs differ between pOCx vectors.
5. Order primers for gRNA and donor.

### (B) Cloning of intermediate construct

1. Digest pOC2 cloning template with BbsI restriction enzyme and purify.
2. Anneal primer pair for gRNA target sequence using a standard primer annealing protocol and dilute to e.g. 20 nM.
3. Ligate primer pair into digested pOC2 (Figure 1-3B). Use, for example, 25 ng of digested backbone and 4 μL of annealed primer pair (20 nM) in a total volume of 20 μL.
4. Transform competent bacteria and screen clones for vectors with correct gRNA target sequence using Sanger sequencing. Sequencing primer: 5’ cgggccatttaccgtaagtt 3’.

### (C) Cloning of final knock-in construct

1. Digest intermediate knock-in construct with HindIII and MluI (or XbaI and MunI).
2. Amplify donor using PCR with the donor primers. While standard PCR protocols generally work well, due to the long primer sequences a touchdown PCR protocol is preferred.
3. Purify digested backbone and correct PCR product (donor).
4. Digest donor DNA with HindIII and MluI (or XbaI and MunI), and purify again.
5. Ligate donor DNA into the digested intermediate construct using a 3:1 molar ratio (Figure 1-3B). Use, for example, 100 ng of the backbone for ligation (20 μL total volume).
6. Transform competent bacteria and screen clones for vectors with correct gRNA target sequence and donor DNA using sequencing. Sequencing primers: 5’ ctagtccgtttttagcgcgt 3’ and 5’ cgggccatttaccgtaagtt 3’.

The resulting pOC2 Cre_off_ knock-in construct can be transfected in the target preparation together with a Cas9 vector to generate knock-ins. Use the BmtI and AfeI restriction sites to exchange the tag of the donor sequence.

### References

Willems J, de Jong APH, Scheefhals N, Mertens E, Catsburg LAE, Poorthuis RB, de Winter F, Verhaagen J, Meye FJ, MacGillavry HD (2020) ORANGE: A CRISPR/Cas9-based genome editing toolbox for epitope tagging of endogenous proteins in neurons. *PLoS Biol* 18:e3000665.
